# Trends and Technological Advancements in the Possible Food Applications of Spirulina and Their Health Benefits: A Review

**DOI:** 10.3390/molecules27175584

**Published:** 2022-08-30

**Authors:** Nawal K. Z. AlFadhly, Nawfal Alhelfi, Ammar B. Altemimi, Deepak Kumar Verma, Francesco Cacciola, Arunaksharan Narayanankutty

**Affiliations:** 1 Department of Food Science, College of Agriculture, University of Basrah, Basrah 61004, Iraq; 2 College of Medicine, University of Warith Al-Anbiyaa, Karbala 56001, Iraq; 3 Agricultural and Food Engineering Department, Indian Institute of Technology Kharagpur, Kharagpur 721302, West Bengal, India; 4 Department of Biomedical, Dental, Morphological and Functional Imaging Sciences, University of Messina, 98125 Messina, Italy; 5 Division of Cell and Molecular Biology, PG and Research Department of Zoology, St. Joseph’s College (Autonomous), Devagiri, Calicut 673008, Kerala, India

**Keywords:** spirulina algae, chemical composition, health and nutritional value, functional foods, food formulation, biological activity

## Abstract

Spirulina is a kind of blue-green algae (BGA) that is multicellular, filamentous, and prokaryotic. It is also known as a cyanobacterium. It is classified within the phylum known as blue-green algae. Despite the fact that it includes a high concentration of nutrients, such as proteins, vitamins, minerals, and fatty acids—in particular, the necessary omega-3 fatty acids and omega-6 fatty acids—the percentage of total fat and cholesterol that can be found in these algae is substantially lower when compared to other food sources. This is the case even if the percentage of total fat that can be found in these algae is also significantly lower. In addition to this, spirulina has a high concentration of bioactive compounds, such as phenols, phycocyanin pigment, and polysaccharides, which all take part in a number of biological activities, such as antioxidant and anti-inflammatory activity. As a result of this, spirulina has found its way into the formulation of a great number of medicinal foods, functional foods, and nutritional supplements. Therefore, this article makes an effort to shed light on spirulina, its nutritional value as a result of its chemical composition, and its applications to some food product formulations, such as dairy products, snacks, cookies, and pasta, that are necessary at an industrial level in the food industry all over the world. In addition, this article supports the idea of incorporating it into the food sector, both from a nutritional and health perspective, as it offers numerous advantages.

## 1. Introduction

Spirulina algae, also known as *Arthrospira platensis*, are members of the class of cyanobacteria (also named blue-green algae) that are classified under the phylum of multicellular organisms. These filaments are unbranched and spiral in shape. Algae are a diverse group of aquatic organisms that have the ability to conduct photosynthesis. In subtropical and tropical climates, such as Hawaii, Mexico, Asia, and Central Africa, they flourish naturally in water tanks that contain high levels of salt and alkaline. GRAS stands for “generally regarded as safe,” which is the designation that the Food and Drug Administration (FDA) has bestowed upon it. Research on humans in clinical trials, as well as studies on animals carried out in the most recent decade, provide credence to this assertion. *A. platensis*, *A. maxima*, and *A. fusiformis* are three of the species of spirulina that have been put to use in food, and have been the subject of a significant amount of research [1,2,3,4].

Spirulina algae have high nutritional value. As a result of their high protein content (60–70% on a dry weight basis), vitamins, minerals, essential fatty acids, and other nutrients, the FDA has designated them as the ideal food for mankind and a “super food,” containing high concentrations of beta(β)-carotene, vitamin B12, iron, trace elements, and the extremely rare essential gamma(γ)-linolenic acid. The Food and Agriculture Organization (FAO) of the United Nations have referred to spirulina as a “highly digestible protein product,” and the US space agency has utilized it as a dietary supplement for astronauts. Because of this, spirulina deserves the title of “the food of the future” more than any other food on Earth [1,2].

It has been demonstrated that spirulina is both biologically and economically significant due to the numerous applications that have been developed for it in the food, pharmaceutical, biofuel, cosmetics, and agricultural industries. These algae are readily accessible for purchase and have a significant geographic distribution. This is because the manufacturers want to obtain the biomass of spirulina in order to make use of its important biologically active compounds, such as phycocyanins, phenols, polysaccharides, polyunsaturated fatty acids (PUFAs), carotenoids, vitamins, and sterols. This is why there is such a high demand for spirulina. The majority of these compounds play an important therapeutic role in the treatment of cardiovascular diseases (CVDs), high cholesterol, high blood sugar, obesity, high blood pressure, tumors, and inflammatory diseases. In addition to bolstering the immune system, the presence of these compounds is associated with a reduced risk of developing neurodegenerative conditions, such as Parkinson’s disease, Alzheimer’s disease, and multiple sclerosis, in particular. Spirulina is regarded as a natural medicine and is utilized in the manufacturing of functional foods and nutritional supplements all over the world due to the qualities that have been described [5,6].

Spirulina algae can be produced in the form of powder, liquid, oil, tablets, or capsules, and are used in many food industries, including the manufacture of sweets, snacks, and pastries. This helps the market meet the demand for variety while also providing highly nutritious food that can aid in the feeding of children and the fight against malnutrition [7]. In addition to the introduction of spirulina in the production of functional beverages, such as fruit juices, which have gained a great deal of relevance in terms of health, it is also employed in the production of dairy products, pasta, oil derivatives, and nutritional supplements [8,9,10]. In addition to being used as a coloring agent in the food industry, spirulina has a wide range of uses in the areas of human nutrition, animal feed, and fish feed [11,12,13].

The production of spirulina algae, which are a rich source of protein, has increased in recent years. This coincides with an increase in the demand for protein, which has led to the development of the industry. Because of this, food companies have begun marketing proteins derived from a range of sources, including those derived from animals, plants, single-celled organisms, and spirulina. Pasta, sushi, and jerky are just a few examples of the new food products that have been produced for consumers that are based on spirulina [14,15].

In spite of the facts on the nutritional, environmental, and social significance of spirulina that have been acquired from a broad spectrum of the published literature, it is still possible to draw the conclusion that the production of spirulina is restricted to a select number of natural places. As a result, a group of researchers and scientists from throughout the world are campaigning for extensive spirulina production everywhere in the world.

The objective of this review paper is to shed light on emerging tendencies and technological developments in a variety of facets of spirulina. This includes providing a concise introduction to spirulina as important algae for human food and health, as well as spirulina’s various nutritional and biochemical components. In addition, this article offers an insightful discussion on the use of spirulina in the food industry for the purpose of the formulation of a variety of food products. The biological and therapeutic significance of spirulina has also received a lot of attention. This includes its importance in weight control, intestinal flora, and immunological activities, as well as its application for the treatment of various diseases such as diabetes, cancer, cardiovascular, and so on.

## 2. A Brief Overview on Spirulina as Important Algae for Human Food and Health

The term “algae” refers to a wide collection of organisms that produce their own food via the process of photosynthesis and may be found in a variety of habitats, including marine and freshwater environments [3]. They are found in almost every part of the world and may be divided into two categories. Microalgae are the most basic and fundamental members of the plant kingdom. The bulk of their cells are rather thin, measuring between 3 and 20 µm, and some species form simple colonies. Macroalgae are typically multicellular, expand at a quick rate, and can reach widths of up to 20 m. When compared to the growth rates of terrestrial plants, the rates of growth of macroalgae are significantly higher. Production of macroalgae in maritime habitats, also known as seaweed, does not need the usage of arable land or fertilizer and can take place without either of those factors being present. Seaweeds have the capacity to generate more biomass per hectare than vascular plants do, develop at a far faster rate, and make use of the light energy and carbon dioxide that is taken in from the environment. In the field of applied botany, the tiny algae known as cyanobacteria were once known as cyanophyceae. Cyanobacteria are some of the earth’s oldest primitives. They are one of the prokaryotes that have certain properties in common with plants, such as the capacity to carry out photosynthesis, and their cytoskeleton is similar (phototrophic nutrition). The cellular forms of cyanobacteria have undergone several transformations during the course of their evolution, ranging from unicellular to multicellular structures. They can be found in ecosystems containing fresh water, marine life, and terrestrial life, as well as certain severe or harsh habitats, such as hot springs, dry soils, some saline environments, and glaciers [3,16,17,18].

*Arthrospira platensis* is the species of spirulina that is multicellular, filamentous, heterogeneous, non-branching, and does not fix nitrogen. It is also capable of photosynthesis and the production of chemical compounds that are necessary for existence. It is grown in liquid farms that are located within open ponds, and flourishes naturally in brackish waters, salt lakes, and warm conditions that are rich in bicarbonate and carbonate [19,20,21,22,23]. In Iraq, many species of spirulina, such as *A. jenner*, were discovered, identified as novel algae, and listed in the inventory of Iraq’s algal flora [24,25].

Before 1962, spirulina was considered to be a type of algae. However, in that year, it was reclassified as a member of the prokaryotic kingdom, and the name “cyanobacteria” was suggested for it [18,26]. Different-sized filaments or spiral trichomes can be produced by organisms belonging to the genus *Arthrospira*. Spirulina may fold and bend to varying degrees, taking on shapes that range from a tightly coiled form to a shape that is straight and unwound. Solitary in nature, filaments reproduce by a process known as binary fission. The lengths of the filaments typically range from 2 to 12 µm but can go as high as 16 µm at times [27,28]. The diameter of the thread ranges anywhere from 3 to 12 µm, and the cells that make up the filament contain gas vacuoles that aid in floating [19,29,30].

## 3. Nutritional and Biochemical Components of Spirulina

**Figure 1:**
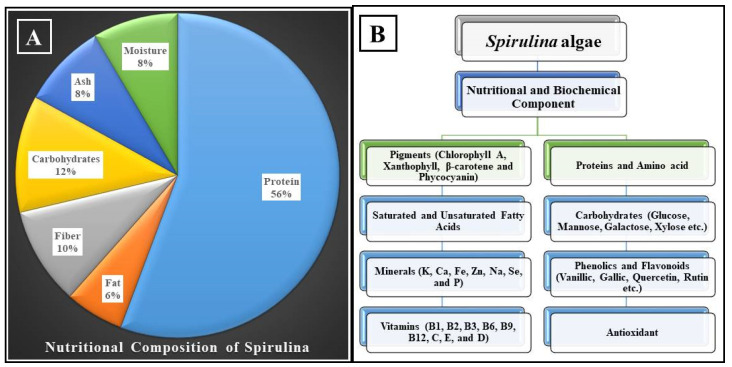
(**A**) Nutritional composition and (**B**) biochemical components of spirulina [36].

Food is the primary means through which the body receives the myriad of vital nutrients that are required for development, the performance of essential biological activities, and the preservation of overall health. On the one hand, considering that our bodies are unable to produce some nutrients, it is necessary to receive them through this diet. On the other hand, several diseases have been related to an imbalance in the human diet, which can be caused by the presence of certain unsuitable nutritional components or the body’s incapacity to absorb them [31]. The overall composition of spirulina changes depending on the source of the algae used to cultivate it, the environmental conditions of the manufacturing facility, and the season of the year. Proteins make up between 55% and 70% of the body of spirulina, while carbohydrates make up between 15% and 25%, fats make up between 6% and 8%, minerals make up between 7 and 13%, moisture (dried algae) makes up between 3% and 7%, and dietary fibers make up between 8% and 10% [32]. Figure 1 presents a description of the components that make up spirulina. The proportion of PUFAs is between 1.5% and 2% of the total fat content, and it is rich in linolenic acid, which accounts for 36% of the total PUFAs, as well as vitamins (B1, B2, B3, B6, B9, B12, C, D, and E) and minerals (K, Ca, Cr, Cu, Fe, Mg, Mn, P, Se, Na, and Zn), as well as the pigments (chlorophyll A, xanthophylls, β-carotene, echinenone, myxoxanthophyll, zeaxanthin, canthaxanthin, diatoxanthin, 3-hydroxychininone, β-cryptoxanthin oscillaxanthin, phycobiliproteins, C-phycocyanin, allophycocyanin) and enzymes (such as lipase) [33]. The components of spirulina’s chemical makeup are summarized in Table 1.

**Table 1:** The value of proximate composition of spirulina from different reported research.

Proximate Composition (%)						Food Energy	References
Moisture	Fat/Lipid	Protein	Ash	Fiber	Carbohydrate		
4–5	4–7	65–72	6–12%	3–7	15–25	2.90 cal/g	[34]
3–7	6–8	55–70	7–13	8–10	15–25	–	[32]
5.37	7.19	61.57	7.10	7.93	16.21	–	[35]
5.45–9.92	6.61–6.84	52.85–65.00	9.55–9.93	9.79–11.37	15.29–13.62	329.89–379.58	[36]
5.27	1.27	71.90	3.50	9.70	13.63	353.55	[37]
4.74	6.93	62.84	7.47	8.12	–	–	[38]
–	7.16	52.95	–	–	13.20	–	[39]
1	6	63	8	–	22	–	[40]
–	4	65	3	3	19	–	[41]
4–6	5–7	55–70	3–6	5–7	–	–	
6	6	61	9	–	14	–	
9	7	60	11	–	–	–	

– Not reported.

### 3.1. Carbohydrates

According to the findings of various studies, the proportion of carbohydrates present in *spirulina* spp. is around 13.6% [42,43]. On the other hand, a number of additional studies came to the conclusion that the total carbohydrate content of spirulina ranged from 15% to 25% dry weight [32,33,42,44,45,46]. There is no cellulose present in spirulina algae’s carbohydrates; instead, they are made up of a variety of sugars, such as glucose, mannose, galactose, and xylose, in addition to glycogen. As a result, the carbohydrates included in spirulina are simple to digest, as well as nutrient-dense, and may be consumed by elderly individuals and those who have intestinal malabsorption. In addition to that, it has a polysaccharide with high molecular weight known as immolina. Rhamnose is the primary component in it, accounting for around 52.3% of the total sugars generated by spirulina. In another variety, rhamnose accounts for roughly 49.7% of the total sugars produced. Spirulina has a biomass of 1.22 g/L, its polysaccharide content is 2.590% of its biomass, and the total sugars that it contains are 17.275% of its polysaccharides [6,43,47,48,49]. Polymers, such as glucosamine (1.9%), rhamnosamine (9.7%), and glycogen (0.5%), as well as small amounts of glucose, fructose, sucrose, glycerine, mannitol, and sorbitol, are the primary components of virtually all absorbable carbohydrates. Spirulina has sugars in its cell wall that are analogous to the sugars found in the cell walls of Gram-negative bacteria. These are composed of glucosamine, muramic acid, and glucosamine that have bound to peptides. Due to the fact that these cell walls are relatively thin, digestive enzymes are able to access the contents of the cell with relative ease [42]. Of the various culture media that are utilized during the production of spirulina, each has an impact on the total amount of carbohydrates that are produced. According to the findings of Madkour et al. [39], the percentage of carbohydrate content in spirulina algae grown in low-cost culture media varied depending on the type of nitrogen source present in the culture medium. To accomplish this, all of the nutrients found in the standard medium are swapped out for more affordable and readily available commercial chemicals and fertilizers in the region. The percentage of carbohydrates present in the medium with the standard nitrogen source was 13.20%, but this percentage increased to 16.01% when the nitrogen source was replaced with a medium containing urea. In the ammonium nitrate (NH4NO3) medium, the concentration of carbohydrates rose to 24.50% on a dry weight basis; however, other researchers discovered that the amount of carbohydrates varied depending on the region of production and the kind of product being made [32].

### 3.2. Lipids/Fats and Fatty Acids

According to the findings of several researchers, the lipid content of *S. platensis* ranges from 5% to 10% of the dry weight. Other research that used more effective extraction techniques found that the percentage was greater than 11%. In most cases, it will contain fats that are necessary for human survival, and free fatty acids will make up between 70% and 80% of the total fat. These total lipids may be divided into a saponified fraction that makes up 83% of the total and an unsaponifiable fraction that makes up 17%, with the unsaponifiable fraction mostly consisting of paraffin, pigments, terpene alcohols, and sterols. Omega-6 fatty acids make up the majority of the total fat, and there is just a trace quantity of cholesterol (less than 0.1 mg/100 g dry mass) present [33,42]. Adults need 1–2% of their total energy intake to come from essential fatty acids, whereas children need 3% of their total energy intake [50].

The location of the closest polyunsaturated point in the MTG is used to describe the optimal omega-6 to omega-3 ratio that some nutritionists advocate, which falls between 4 and 5 [31,51]. It was discovered that the total fatty acid concentration of *A. platensis* is 81.2 mg/g on a dry weight basis, which demonstrates that spirulina is an excellent source of fatty acids [52]. However, Sharoba [38] discovered that the proportion of total saturated fatty acids was 44.21 mg/100 g, but the proportion of total essential unsaturated fatty acids was 55.79 mg/100 g. When looking at the nutritional value of spirulina, researchers found that it has a significant amount of palmitic acid (16:0), which makes up more than 60% of the lipids in *S. maxima* and 25% in *S. platensis*, respectively. While the proportion of saturated palmitic acid in the total fatty acids was 25.8%, the percentage of γ-linolenic acid in the total fatty acids was 40.1%. Spirulina is an excellent dietary supplement for essential fatty acid deficits as a result [42].

Spirulina was discovered to have a significant quantity of PUFAs, with levels ranging from 1.5% to 2.0% fat. This has piqued the curiosity of many researchers, who have been doing studies on PUFAs to determine how much of this nutrient is contained in spirulina [23]. According to the findings of another study, PUFAs made about 30% of the total fats [6], while other researchers reported that the proportion of these fatty acids ranged between 19.4% and 21.9% of the total fatty acids [53]. Its primary fatty acid, 15,12.9-octadecatrienoic acid, accounted for 10.1% of its total fatty acid content, whereas the omega-3 content accounted for less than 1% of its total fatty acid content. Additionally, it had some omega-6 type fatty acids. In addition to this, a significant amount of saturated hexadecanoic acid was discovered (37.6%). The concentration of monounsaturated fatty acids (MUFAs) was low, with the octadec-9-enoic acid (18:1) omega-9 type falling below 2.0%. The quantity of γ-linolenic acid, which came in at 16 mg, had the greatest content, followed by palmitic acid, which had the highest percentage (23%), while myristic acid had the lowest percentage (0.2%) [52].

According to Matos et al. [53], the amount of fatty acids in spirulina algae might vary depending on a variety of parameters, including the growing circumstances and development stage at the time of harvest. The total fatty acid content was estimated to be 4.25 mg/100 g, and it was discovered to include sapienic acid at a level of 2.25 mg/100 g, linoleic acid at a level of 16.7%, and γ-linolenic acid at a level of 14% [51]. According to the findings of Alyasiri et al. [54], one gram of spirulina has a high concentration of linolenic acid of the omega-6 type; specifically, the concentration was 29.1 mg/g, which corresponds to a rate of 2.91%. Additionally, it has PUFAs, which are saturated with 18 carbon atoms and include omega-6. When it comes to the most significant biologically active compounds found in spirulina, phytol had the highest percentage (100%), followed by monolinoleoylglyceroltrimethylsilylether-1b (71.31%), steroid and cholestan-3-ol (2-methylene-3β, 5α) (54.62%), and 9, 12, 15-octadecatrienoic acid, 2, 3-dihydroxypropyl ester (28.21%), hexadecanoic acid methyl ester (23.23%), and methenamine (23.21%) [55]. According to Legezynska et al. (2014), spirulina algae are one of the primary sources of omega-3 fatty acids that fish feed on. Examples of these fatty acids are docosahexaenoic acid (DHA) and eicosapentaenoic acid (EPA). As a result, a higher percentage of these essential fatty acids might be found in fish oils [56]. Linoleic acid, which belongs to the omega-6 group, and alpha (α)-linolenic acid, which belongs to the omega-3 group, is found in the fats of marine algae, suspended algae, and fish oils, respectively. EPA, DHA, α-linolenic acid, and docosapentaenoic acid are the four essential fatty acids (omega-3) that are considered to be of the utmost importance [57]. According to the research conducted by Liestianty et al. [52], the fatty acids contained in spirulina include myristic, heptadecanoic, stearic, oleic, palmitoleic, omega-3, omega-6, linoleic acid, and palmitic acid. Omega-6 kinds, the most significant of which are palmitoleic, oleic, linoleic, and γ-linolenic, and omega-3 types, including α-linoleic acid, are among the most essential types that may be found [38]. Linolenic acid, stearidonic acid, EPA, DHA, and arachidonic acid are found in high concentrations in it [23]. The omega-6 family, which includes γ-linolenic acid and arachidonic acid, and the omega-3 family, which includes EPA and DHA, are the most essential long-chain PUFAs that algae can produce [6]. Utilizing gas chromatography–mass spectrometry (GC-MS) and high-performance liquid chromatography (HPLC), Al-Dhabi and Valan Arasu [51] were able to identify PUFAs in 37 different commercialized spirulina species. Myristic acid, stearic acid, and eicosadienoic acid were identified as the three saturated fatty acids that were present in the spirulina samples. It was found that ten of the unsaturated fatty acids in the spirulina samples were substantially different from one another.

The accumulation of toxic compounds in fish, as well as the odor, strange taste, and oxidative instability of the oils extracted from fish, has a negative impact on the total dependence on the synthesis of long-chain PUFAs (especially omega-3 type) from fish oil. This has a negative impact on the total dependence on the synthesis of long-chain PUFAs from fish oil. As a result, the focus shifted toward the possibility of employing spirulina algae in a commercial environment as a different source to produce these fatty acids [56].

Spirulina algae are a potential source of polyunsaturated fatty acids (PUFAs). Essential fatty acids, such as omega-3 and omega-6, are unable to be produced by humans and, as a result, must be received through the consumption of food. They play a significant role in preserving health and warding off disease. Even though the human gut microbiota is capable of synthesizing long-chain fatty acids, such as linoleic and α-linolenic acids, the synthesis of these acids is controlled by various variables, which makes the consumption of these fatty acids vital for the maintenance of good health [6]. Because it is not commonly found in foods that people eat on a regular basis, despite it having a high nutritional value, the presence of -linolenic acid is interesting. This acid is typically generated in humans from γ-linolenic acid (18:2 omega-6), which comes from vegetable sources [42].

Spirulina is the only food source that contains large amounts of essential fatty acids, especially γ-linolenic acid, which is an omega-6 type that helps regulate all hormones and has anti-inflammatory properties. Comparatively, breast milk is the only food source that contains large amounts of essential fatty acids [19,39,54]. The other supply comes from the oil that is derived from borage, black currant, and evening primrose seeds. In comparison, an evening primrose oil intake of 500 mg has just 45 mg of γ-linolenic acid, whereas 10 g of spirulina has 135 mg of γ-linolenic acid. Comparatively, evening primrose oil only contains 9% linoleic acid, whereas the lipids of spirulina contain around 20–25% of γ-linolenic acid [58].

### 3.3. Protein

The structure and function of the body, as well as the organization of tissues and organs, are all significantly impacted by the presence of protein. If all of the essential amino acids (EAAs) are present in the food that is ingested, the body will be able to generate the protein that it requires. A protein is considered to be of excellent quality if it includes all of the essential amino acids (EAAs) in the amounts required by the body while retaining its bioavailability. The fundamental components of life are referred to as proteins and amino acids. When comparing various sources of protein, the following criteria should be taken into account: the quantity of protein, the quality of the amino acids, the amount of protein that can be consumed, the ease with which the protein can be digested, and the amount of fat, calories, and cholesterol that the protein contains [57].

Spirulina has a protein level that is quite high, reaching from 60% to 70% of its dry weight (compared to 22% in beef). This type of algae contains an extraordinarily high amount of protein for a plant source; in fact, it contains twice as much protein as the finest source of protein found in vegetables. This amount is significantly higher than the percentages found in animal meat and fish (15–25%), soybeans (35%), powdered milk (35%), peanuts (25%), eggs (12%), cereals (8–14%), and whole milk (3%). Table 2 compares the amount of protein found in various dietary sources to that which may be found in spirulina algae. The amount of protein that these algae contain fluctuates by between 10% and 15% depending on the time of harvest, with the most protein being present in the algae during the early morning [42,57].

Spirulina is a great food source of proteins because it has a high percentage of essential amino acids (EAAs), which account for around 38.81–47.00% of the total weight of proteins. The quantity, proportion, and quality of the amino acid contents of a protein are used to evaluate the protein’s overall quality [2,42]. Leucine, valine, and isoleucine are the three amino acids in spirulina that have the greatest concentrations, and complete spirulina proteins include all of the EAAs. Spirulina is superior to all plant proteins, including legume proteins, despite having lower concentrations of the amino acids methionine, cysteine, and lysine than the conventional dietary proteins that come from meat, eggs, or milk [33,45].

Undernutrition is a problem that affects public health, particularly in developing nations. This has led to a trend toward the utilization of spirulina algae, which has been utilized as a functional food for decades. Developing countries are more at risk of undernutrition. According to Salmeán et al. [79], spirulina has adequate sensory properties and has not shown any sign of toxicity, which means that it is safe for human ingestion [79]. Because it has such a high proportion of macro- and micronutrients, this algae was initially utilized in the health food and nutritional supplement sector as a protein supplement. In many countries outside Europe and North America, it is often referred to as supplementary food [23,48,80,81]. According to the findings of El-Chaghaby et al. [82], the protein content of spirulina platensis was 53.30% (dry weight), which was much higher than that of Chlorella vulgaris (20.67%) and Scenedesmus obliquus (31.07%).

Batista et al. [83] were able to boost the protein availability in the biscuit samples by adding *A. platensis* as a high protein food source. On a dry weight basis, *A. platensis* is composed of 68.9% protein; therefore, this allowed them to achieve their goal. The greatest levels of IVPD (in vitro digestibility protein) were reported in samples that included 6% algae. These samples contained 14.3% protein and had IVPD values of 83%. The quantity of protein that could be digested in the treated samples was 11.9 g/100 g of biscuits. This proportion is much greater than the one found in the control sample, which had a protein content of 9.8% and a digestible protein content of 7.3 g/100 g of biscuits. Protein insufficiency is a prevalent condition that affects more than 300 million individuals throughout the world [79]. Protein deficiency may be treated, but it requires a dependency on the ingestion of protein from various sources.

The plant proteins known as phycobiliprotein, which are C-phycocyanin and allophycocyanin in a ratio of around 10:1, are responsible for the majority of spirulina’s beneficial effects on human health [32,45]. C-phycocyanin is one of the primary proteins that may be discovered in moss, and it accounts for around 20% of the total dry weight of all protein fractions. This particular pigment is a molecule that is similar to biliverdin in that it contains phycocyanobilin [3,30,43,79,84].

Proteins included in spirulina are not difficult to digest, even for elderly individuals who have difficulties absorbing complex proteins via their intestines and who adhere to certain diets. This is because the cell walls of algae are composed of mucopolysaccharides, which are simple sugars that are easy to digest and absorb. Cellulose, on the other hand, is indigestible for humans. Additionally, it has a high digestibility of its proteins, ranging from 85% to 95%, and the process of acquiring proteins through proteolytic enzymes is straightforward in comparison to the process of obtaining enzymes from vegetables. Patients suffering from malnutrition, such as kwashiorkor, in which there is a reduction in the capacity of the gut to absorb nutrients, would be good candidates for this treatment. It was discovered that this algae is more beneficial in children who are suffering from malnutrition than milk powders, which contain lactic acid that is difficult to digest and absorb. This alga was shown to be more effective than milk powders [3,43,57,85].

### 3.4. Amino Acids

Proteins are composed of amino acids, which are the core structural units of proteins and also the fundamental components of numerous coenzymes, hormones, and nucleic acids. Foods that include all of the EAAs are essential for overcoming a wide variety of dietary and health issues, since these EAAs play a variety of structural and functional roles in the body. It is essential to have access to a sufficient amount of protein in one’s diet in order to preserve the structure and function of one’s cells, as well as one’s health and capacity [10]. There is a correlation between the presence of EAAs and the quality of proteins. Animal proteins are the only source of complete proteins and are an abundant source of EAAs, which the human body is unable to produce on its own for biological reasons. Plant proteins are considered to be incomplete proteins because they are missing one or more of the EAAs. These essential amino acids include histidine, isoleucine, lysine, methionine, phenylalanine, threonine, and valine [12].

Proteins derived from spirulina have a comprehensive profile thanks to the presence of EAAs and non-EAAs in enough quantities in each molecule. It has EAAs, which account for 47% of the algae’s total protein weight, so it is really good. These include leucine, tryptophan, methionine, phenylalanine, lysine, thionine, and valine; the corresponding levels of these amino acids were 55, 10, 14, 28, 30, 33, 36, and 45 mg/g. In terms of the non-EAAs, they contribute to the synthesis of the proteins that are required by the cells of the body alongside the EAAs, which is how they play a vital part in the body. Any non-EAAs that are present in excess can be turned into glucose, which serves as a source of energy for the body. In general, it was discovered that the non-EAAs contained in spirulina include cysteine, histidine, proline, tyrosine, glycine, serine, arginine, alanine, aspartic, and glutamate acid at levels of 7, 10, 27, 30, 32, 33, 44, 47, 60, and 92 mg/g, respectively. These amino acids were detected in spirulina [42,52].

Spirulina contains the amino acids methionine, lysine, threonine, tryptophan, isoleucine, leucine, phenylalanine, valine, alanine, arginine, cysteine, glutamine, glycine, histidine, proline, serine, and threonine [46]. According to research conducted by Siva et al. [18], the highest levels of the EAAs leucine and valine were found in spirulina at 5400 and 4000 mg/100 g, respectively. On the other hand, the highest levels of glutamic acid and aspartic acid, which are not EAAs, were found at 9100 and 6100 mg/100 g, respectively. Salmeán et al. [79] reported that EAAs were found in the highest amounts of leucine (5380 mg/100 g), valine (3940 mg/100 g) and isoleucine (3500 mg/100 g), while the non-EAAs were reported as glutamic acid, aspartic acid, and alanine with values of 9130 mg/100 g, 5990 mg/100 g, and 4590 mg/100 g, respectively.

Isoleucine, leucine, lysine, valine, arginine, alanine, aspartic acid, glutamic acid, and glycine were the amino acids that were discovered in dry spirulina algae; their quantities were 3.209, 4.947, 3.025, 3.512, 4.147, 4.515, 5.793, 8.386, 3.099 g/100 g, respectively [43]. According to the research conducted by [86], proteolytic enzymes were utilized in order to extract the amino acids from dry extracts of spirulina algae. It was discovered that the extracts were abundant in free amino acids as well as short peptides. Furthermore, it was discovered that the Alcalase enzyme extract contained the highest percentage of amino acids, which was 45% (weight-to-weight) dry extract, whereas the extract without enzymatic aid produced only 34%. This value was in line with the range of amino acid content in spirulina, which was between 50% and 65% (weight-to-weight). The quantity of amino acids that could be extracted from spirulina using the Alcalase enzyme approach was 1426 μmol/g, while the amount that could be extracted using the traditional method was only 573 μmol/g [86].

Because of the high nutritional value of EAAs, their application in the production of nutritional supplements has become more widespread in recent years. EAAs make up around 35% of the total amino acids found in spirulina [6]. Due to the fact that spirulina and its extracts include a diverse assortment of EAAs and non-EAAs, they can serve as a source of nutrients and nutritional supplements. It was discovered that glutamic acid and aspartic acid are the most prevalent non-EAAs found in dry algae, whereas isoleucine and phenylalanine are the most common EAAs. Glutamic acid was shown to be the most abundant of the three. The level of the amino acid arginine was found to be greater in the bulk of spirulina, with 8.153 µmol/100 mg, compared to the extract, which had 7.89 µmol/100 mg of dry weight. It was discovered that the biological value of the proteins in spirulina is very high, complete, and contains all of the EAAs, despite having a low percentage of methionine and cysteine when compared to the standard proteins egg albumin and milk casein. This was discovered when comparing the proteins of spirulina to those of egg albumin and milk casein. However, according to Salmeán et al. [79], the proteins found in spirulina are of better quality than the proteins found in any other vegetable protein source, including legumes and soybeans.

Fortifying foods with spirulina, which leads to an increase in the amount of amino acids in treated foods, has been shown to be beneficial by a significant number of researchers who have published their findings. This has a beneficial impact on increasing the nutritional content of foods that have been fortified. Spirulina in its dry form is an excellent source of amino acids, particularly the amino acids alanine, arginine, aspartic acid, glutamic acid, leucine, and valine, which are the most plentiful. The addition of spirulina to biscuits at several levels (0%, 5%, 10%, and 15%) was the subject of an experiment, and the results showed that the amount of amino acids present in the finished product grew as the percentage of addition increased [34]. Aljobair et al. [10] found that the addition of spirulina to two different types of date juice boosted the amount of essential amino acids (EAAs). The total amount of EAAs found in spirulina algae, as well as in two other types of date juice that were supplemented with spirulina at a concentration of 10%, were, respectively, 38.46%, 48.69%, and 46.02%. In addition, spirulina has a high proportion of total non-EAAs; specifically, 61.54%, which is followed by date juice fortified with 10% spirulina, which has 53.98% and 51.32% respectively. In comparison to the proteins found in dates, spirulina algae proteins are of a far higher quality, which contributes to the juice’s high concentration of amino acids.

### 3.5. Vitamins

Vitamins are necessary micronutrients, but since humans are unable to produce them in adequate quantities, they need to be received from the food that they eat. Their absence is linked to a wide variety of ailments, and foods derived from algae are particularly rich in vitamins [3,81]. Of these algae, spirulina is employed in the development of functional foods. As an alternative source of vitamin production, it generates a significant quantity of spirulina at an affordable price [86]. It was demonstrated that spirulina contains a high concentration of vitamins, and the addition of these algae to food products, such as drinks and juices, can boost the vitamin content of the product while also improving its nutritional and health benefits [10]. These algae contain all vitamins, including vitamin A (β-carotene), vitamin D, vitamin E, vitamin K, vitamin C, and vitamin B complexes, such as thiamine (vitamin B1), riboflavin (vitamin B2), niacin (vitamin B3), pantothenic acid (vitamin B5), pyridoxine (vitamin B6), folic acid (vitamin B9), and cobalamin (vitamin B12) [6,10,32,85,86]. Edelmann et al. [87] investigated the amount of various vitamins that are present in dry spirulina algae. These vitamins include riboflavin (vitamin B2), cobalamin (vitamin B12), and folic acid (vitamin B9), and their respective amounts were 36.3, 2.4–0.6, and 3.5 μg/g, while the amount of niacin (vitamin B3) was 0.16 mg/g. The researchers came to the conclusion that the dry spirulina had a low quantity of folic acid and that the majority of vitamin B12 was inactive. Therefore, they suggested that spirulina powder in the amount of 4–5 g per day is what one ought to consume on a daily basis in order to fulfill one’s needs for folic acid and vitamin B12 [87]. According to Choopani et al. [31], dried spirulina includes a wide variety of vitamins, the levels of which range from 100–200, 1.5–4.0, 0.5–0.7, and 5.0–20 mg/100 g, respectively, for vitamins A, B1, B6, and E, respectively.

According to the findings of a number of studies that referred to the analysis of vitamins, dried spirulina contains 3.6 mcg/g of vitamin B12 and is an excellent source of β-carotene with a content of 5.8 mg/g. β-carotene is absorbed by the body and transformed into vitamin A. Consuming between 1 and 2 g of spirulina per day is adequate to fulfill the body’s daily requirements for vitamin A, which are around 1 mg/day [52]. It is crucial to remember that 100 g of spirulina includes 1100 IU of vitamin A, which is necessary for maintaining healthy immunity, eyesight, and reproduction [86]. It was described that dried spirulina is abundant in beta-carotene, which makes up nearly half of the carotenoids, and 1 g of spirulina has 0.9 mg of all-trans β-carotene. It was also reported that spirulina includes a significant amount of vitamins, including vitamin A (β-carotene) at 211 mg/100 g, vitamin K at 1090 µg, and vitamin B12 at 162 µg [88]. According to Seyidoglu et al. [43], dry spirulina is an excellent source of vitamins, such as vitamin E, vitamin B1, vitamin B2, vitamin B3, vitamin B6, vitamin B12, vitamin K, folic acid, biotin, and pantothenic acid. The amounts of these vitamins in dry spirulina were 5, 3.5, 4.0, 14.0, 0.8, 0.32, 2.2, 0.01, 0.005, 0.1 mg/100 g. According to Stanic-Vucinic et al. (2018), 3 g of spirulina contains 11,250 IU, 75 µg, and 9 µg of vitamin A, vitamin K, and vitamin B12, respectively. Spirulina is a good source of these vitamins. A sample of 10 g of spirulina contains vitamin A (23000 IU), vitamin B1 (0.35 mg), carotene (14 mg), vitamin B2 (0.40 mg), vitamin C (0.8 mg), vitamin B3 (1.4 mg), vitamin D (1200 IU), vitamin B6 (60 mg), vitamin E (1.0 mg), folic acid (1.0 mg), vitamin K (200 mg), vitamin B12 (20.0 mg, biotin (0.5 mg), pantothenic acid (10.0 mg), and inositol (6.4 mg) [32]. It was demonstrated by Falquet and Hurni [42] that the β-carotene found in spirulina can be converted into vitamin A in mammals. The daily requirement for vitamin A in mammals is estimated to be less than 1 mg, and it only takes 1–2 g of spirulina to fulfill this requirement [79]. Spirulina in its dry form also has vitamin E (190–50 mg/kg), which is enough to meet the recommended daily allowance of 12 mg. Spirulina is enriched with vitamin B12, a nutrient that is hard to come by in a vegetarian or vegan diet because it is only found in animal products. It is essential for the production of red blood cells as well as DNA, and it is present in quantities that are four times higher than those found in raw liver [6,86,89]. In addition to that, it has the bioavailable form of vitamin B12 known as methylcobalamin, which is found in it. Spirulina, which has 35–38 μg/100 g, is regarded as the food that contains the highest concentration of this vitamin [33]. Because the daily needs of vitamins B1, B2, B3, and B12 are met by consuming 20 g of spirulina, it is considered a complete source of these vitamins [45]. Mogale [90] found that the vitamins found in spirulina algae play an important role in the metabolism of cells. Additionally, he found that the vitamin A, vitamin E, and vitamin C present in the aqueous extract of spirulina are nonenzymatic antioxidants that protect membrane lipids from oxidative damage. They had a vitamin A content of 18.1 mg/g, a vitamin E content of 3.91 mg/g, and a vitamin C content of 17.2 mg/g, whereas the recommended daily allowances for these vitamins are 0.9 mg/day, 90 mg/day, and 15 mg/day, respectively [54].

There are various environmental variables (cultivation conditions), harvesting methods, and cell drying methods that can have a significant impact on the vitamin content of microalgae [6]. These factors can also substantially modify the vitamin content of microalgae In addition to this, it was discovered that the process of extracting vitamin B12 from spirulina algae has an impact on the total quantity of vitamin that is produced, There were six different extraction procedures employed. The extraction using potassium cyanide (KCN), which recovered 92–95% of the vitamin, was shown to be the most effective approach [91]. Rats were given a B12-deficient diet for the duration of six weeks in a comparative study that was carried out by Usharani et al. [23]. The rats were also given spirulina as a source of vitamins to supplement their diet. Rats given a diet consisting of spirulina had a significantly increased amount of cobalamin in their livers. El-Nakib et al. [34] also found that an increase in the proportion of algae led to an increase in the number of vitamins present in enriched biscuits (0%, 5%, 10%, and 15%). Dry spirulina has significantly higher vitamin content than other food sources, such as liver, carrots, spinach, and vegetables. Aljobair et al. [10] came to the conclusion that enriching date juice with spirulina led to an increase in the quantity of vitamins already present in the juice as well as a reduction in the deficiency of vitamins, such as vitamin B3, vitamin B5, vitamin B6, vitamin B12, vitamin E, and vitamin K in the juice. This was one of the main findings of the study.

### 3.6. Minerals

Spirulina is an excellent source of a wide variety of minerals, such as potassium (K), calcium (Ca), chromium (Cr), copper (Cu), iron (Fe), magnesium (Mg), manganese (Mn), phosphorous (P), selenium (Se), sodium (Na), boron (B), molybdenum (Mo), and zinc. Other nutritional components include boron (B), phosphorous (P), and selenium (Zn). It also has a very high proportion of both macro- and micronutrients, in addition to other nutritious components, and its products are utilized in agriculture, the food industry, the pharmaceutical industry, the perfumery industry, and medical practice [23,33]. The quality of the nutritional supplement offered by two different species of spirulina, *A. platensis* and *A. maxima*, was analyzed, and the mineral components were identified for each of the four concentrations [92]. Using microwave-induced plasma atomic emission spectroscopy, the levels of 15 elements that were found in 11 different dietary supplement products were investigated and studied. These elements included Al, Ba, Ca, Cd, Cr, Cu, Fe, K, Mg, Mn, Na, Ni, P, V, and Zn [92]. In all of the analyses conducted, the results showed that the levels of the mineral elements in the spirulina samples fell below or were within the level of the recommended daily intake (mg/daily) that was established by the Codex Alimentarius Commission (CODEX). This information was provided by the FDA. However, there was an exception for the concentration of Cd, which was beyond allowed limits; this was explained by the capacity of algae to bioaccumulate this element. The Cd concentration was above permissible values.

The Fe content in spirulina is 10 times higher than that of other foods that are rich in Fe. Spirulina is an iron-rich diet. Spirulina’s Fe content is absorbed by the body at a rate that is approximately 60% higher than that of ferrous sulfate, which is often included in Fe supplements. Due to the high percentage of Fe in spirulina and the relevance of Fe in the treatment of Fe deficiency (anemia), many studies have been conducted on the topic. Pregnant women and children are particularly at risk of Fe insufficiency. It was revealed that it does not cause any toxicity in comparison to Fe supplements that are administered in the form of ferrous sulfate, which is known to produce toxicity issues and frequently leads to diarrhea. Additionally, cereals have a high concentration of phytic and oxalic acids, both of which greatly inhibit the bioavailability and absorption of Fe (as happens in spinach). For spirulina, the bioavailability of Fe was proven in both people and rats. Many dietitians indicate that most food sources have relatively low ratios of K to Na; however, spirulina has a high K content. Spirulina contains Ca, P, and Mg in proportions that are comparable to those found in milk. Because of this, there is no danger of decalcification, which can occur when the amount of P in a food source is increased [33,42]. According to Salmeán et al. [79], the Fe content of spirulina algae is roughly 580–1800 mg/kg, which is a reasonably significant amount when compared to cereals, which are a rich source of Fe and contain 150–250 mg/kg [79].

According to the findings of El-Nakib et al. [34], the incorporation of spirulina during the production of biscuits led to an increase in the amount of minerals present, hence improving the products’ overall nutritional value. This is because attending school has been shown to improve the health of children who are undernourished. The same study found that the Fe concentration of spirulina, which is 0.522 mg/g, is greater than the Fe content of spinach, which is 0.109 mg/g, and the Fe content of soybeans, which is 0.115 mg/g. The amount of Ca, Mg, Fe, P, K, and Na that was present in dry spirulina was 168, 2.55, 0.52, 9.18, 18.30, and 10.98 mg/g, respectively; meanwhile, the amount of Mn, Zn, B, Cu, Mo and Se that was present in spirulina was 19, 2, 30, 3, 30, and 5 g/g, respectively. According to Verdasco-Martín et al. [93], the percentage of minerals found in spirulina is 15%, and the most important minerals are Fe, Ca, P, and K. These minerals are significant and necessary for the construction of the body as well as the performance of its numerous essential functions [94]. When researching the role of spirulina as a nutritional supplement, Siva et al. [18] found that the algae contained minerals that were within the recommended dietary allowance (RDA). These minerals included Ca (1300 mg), Fe (10 mg), iodine (150 mcg), P (700 mg), Mg (420 mcg), Zn (11 mg), Se (0.055 mg), Cu (0.9 mg), Mn (2.3 mg), B (1000–10,000 mcg), and germanium (1.5 mg). The majority were within the range of 100 g of spirulina, although Cr (35 mcg) and Mo (45 mcg) were safe with 10 g/day of spirulina consumption (DRIs, 2004). According to the findings of a number of studies, the most important minerals found in spirulina are Fe, Ca, P, and K, with percentages of 0.058–0.18%, 0.13–1.4%, 0.67–0.9%, and 0.64–1.54% of the dry weight, respectively [45].

Spirulina contains all of the necessary minerals, including Ca, K, Mg, Na, P, Cu, Fe, Mn, Zn, Cr, Se, B, and Mo, which may be obtained in the following proportions: 922.278, 2085.28, 1.1902, 1540.46, 2191.71, 1.2154, 273.197, 5.6608, 3.6229, 0.325, 0.394, 0.325, and 0.394 mL/g [39]. Spirulina is a nutritional supplement that is appropriate for vegetarians, because it is able to absorb the mineral elements that are present in the culture medium in high concentrations while it is growing. These elements include Fe, Ca, P, and K in the following proportions: 0.58–1.8, 1.3–14, 6.7–9.0, and 6.4–15.4 g/kg, respectively [33]. It was revealed that the quantity of minerals in dried spirulina algae is as follows: 700 mg/100 g for 700 mg/100 g for Ca, 0.28 mg/100 g for Cr, 1.2 mg/100 g for Cu, 100 mg/100 g for Fe, 400 mg/100 g for Mg, 5.0 mg/100 g for Mn, 800 mg/100 g for P, 1400 mg/100 g for K, 900 mg/100 g for Na, and 3 mg/100 g for Zn [43]. The mineral composition of spirulina is determined by the source of the algae used to make it as well as the conditions under which it was grown [28,33]. The production of spirulina algae, which may absorb heavy metals if they are present in the culture medium, has been shown to raise concerns about the presence of heavy metals, which has been reported to be a reason to be concerned. Therefore, it is utilized in the removal of heavy metals from contaminated water in the cultivation of spirulina. According to EU Commission Regulation (EC) 1881/2006 for the maximum levels of some pollutants in foodstuffs, the allowable amounts of lead (Pb), Cd, and mercury (Hg) for food supplements made from algae are 3, 3, and 0.1 mg/kg, respectively. This regulation establishes the maximum levels of certain pollutants that can be present in foodstuffs.

### 3.7. Pigments

One of the greatest dietary sources rich in pigments are spirulina algae, particularly C-phycocyanin, which has 14% of the element Fe in it. Additionally, it has the greatest value of chlorophyll (1%) Chlorophyll A pigment is a type of phytonutrient that assists the body in cleansing and detoxifying itself [43]. Spirulina included chlorophyll and phycocyanin pigments, both of which are potent antioxidants; the percentages of these pigments were 1.472% and 14.18%, respectively [2]. Researchers from all around the world are interested in examining the efficacy of these pigments, as well as their uses, applications, and quantitative effects. It was confirmed that the alcoholic extract of spirulina algae contains many important pigments. These pigments include chlorophyll and β-carotene, as well as the protein pigments phycocyanin, allophycocyanin, and phycoerythrin, with their quantities being, respectively, 0.301 mg/g, 0.372 mg/g, and 0.247 mg/g. This is due to the fact that the majority of the antioxidant activity comes from these pigments [95]. There are three primary categories for the pigments found in phycobiliproteins. The primary pigment is called C-phycocyanin, and it accounts for around 20% of the dry weight of the substance. It is also sold in the commercial world as an antioxidant and an anti-inflammatory agent, in addition to its usage as a natural colorant. Phycoerythrins are water-soluble protein compounds that have many beneficial effects on one’s health [45,96,97,98,99]. Phycoerythrins and allophycocyanins are typically present in lower amounts in a 1:10 ratio.

Spirulina contains a number of essential plant pigments, including total carotenoids (400–650 mg/100 g), β-carotene (150–250 mg/100 g), xanthophylls (250–470 mg/100 g), zeaxanthin (125–200 mg/100 g), chlorophyll (1300–1700 mg/100 g), and phycocyanin (15,000–19,000 mg/100 g) [89,100]. It was also reported that the overall concentration of carotenoids in dried spirulina was 6.928 mg/kg, with the total content of xanthophylls accounting for 83.6% (5.787 mg/kg) of the total carotenoid content [43,101]. The levels of phycocyanin, chlorophyll, and carotene found in spirulina were measured and found to be 180, 11, and 6 mg/g of spirulina, respectively [52]. Phycocyanin is a member of an important group of pigments that are found in spirulina algae. Patel et al. (2005) conducted research on the quantitative assessment of the phycobiliprotein pigments C-phycocyanin, allophycocyanin, and phycoerythrin in three distinct species of cyanobacteria. These cyanobacteria species were *spirulina* spp. and *Phormidium* spp. and *Lyngbya* spp. The amounts of phycocyanin pigment were 17.5%, 4.1%, and 3.9% weight to weight for the three different algae, respectively. The amounts of allophycocyanin pigment were 3.8%, 1%, and 0.8% weight to weight, while the quantity of phycoerythrin pigment was 1.2, 0.3, and 0.4% weight to weight. Usharani et al. [23] also reported that spirulina algae have a variety of photosynthetic pigments, including chlorophyll A, xanthophyll, β-carotene, echinenone, myxoxanthophyll, and phycobiliprotein pigments, in addition to lutein, zeaxanthinoxant echinenone, and β-cryptoxanthin pigments [43,101].

Protein pigments are responsible for converting the light energy that falls within the visible wavelength range, which ranges from 400 to 700 nanometers, into chemical energy [102,103,104,105]. At a pH of 7, phycocyanin had the greatest amount of solubility; however, the protein quickly denatures at a pH lower than 3, which causes the color to precipitate [48,102]. It has been shown that the use of an ultrasonic water bath is the most effective way for extracting phycocyanin from spirulina algae. This method produced 43.75 mg/g of pigment at a concentration of 0.21 mg/mL [106]. There are a few different approaches that may be taken to accomplish this task.

Pigments are sensitive to a wide variety of environmental conditions, including temperature, light, oxygen, pH, and oxidizing agents, such as ascorbic acid and trace metal ions, among others. C-phycocyanin is a protein pigment that is photosensitive, heat-unstable, and vulnerable to the oxidation caused by free radicals [48]. Stability in the pigment is provided by the sugars glucose (20%), sucrose (20%), or sodium chloride (2.5%). The acids citric and benzoic can further reduce the heat degradation rate of the pigment [103,107,108]. Abd El-Monem et al. [103] conducted research to investigate how the pigment content was affected by the pH function. The highest pigment content was found at pH 10, with a concentration of 2.8 μg/mL chlorophyll A and 2.6 μg/mL carotenoids, whereas the highest pigment stability was found at pH 5–6 [103,109]. The impact of medium salinity on the pigments was also tested at concentrations of 15, 20, 25, and 30 parts per thousand (ppt), and it was seen from the quantity of chlorophyll A and phycobiliprotein pigments that salinity did not influence these pigments [110]. It was discovered that the lowest possible light intensity resulted in the best production of pigments when exposed to 80 µmol/m^2^/second. This was the case regardless of the influence that light intensity had on the exposure length. On the other hand, photodegradation of pigments happens by photooxidation when exposed to very intense light intensity over an extended period of time. The amount of phycocyanin that was present increased as the light intensity rose. The light had an intensity of 160 µmol/m^2^/second. Once more, the production of pigment was made significantly better. The longer the darkness lasted, the higher the pigment production as well as the amount of energy and biomass that was produced [111]. Sandeep et al. [59] conducted research on the influence that the type of culture medium has on the quantity of pigments. They discovered that the contents of phycocyanin pigment in the treated seawater medium and the m-NRC standard medium were comparable, coming in at 50.9 and 50.95 mg/g, respectively, but that it dropped to 49.82 mg/g in the mixture medium consisting of a combination of m-NRC standard medium and mixture medium (treated seawater: shrimp wastewater). When compared with the standard medium, the content of total carotenoids in the mixed medium was 20.3% lower than what it was in the standard medium. The chlorophyll pigment level was comparable between the two media.

### 3.8. Phenols and Flavonoids

Phenolic chemicals, also known as polyphenolics and present in abundant amounts in algae, are often regarded as being among the most significant naturally occurring antioxidant molecules. They are by-products of the metabolism and are associated with the systems of chemical protection mechanisms that algae have against a variety of biological stimuli, such as ultraviolet rays, pathogens, and mineral pollution [6]. In general, phenolic acids make up one-third of the phenolic compounds, while flavonoids make up the remaining two-thirds. Flavonols and anthocyanins make up the majority of the flavonoid compounds that are found in the diet. Among the phenolic compounds found in algae is the fluorotannin compound, which is primarily found in brown algae, but can also be found in some red algae in smaller quantities. This compound participates in the formation of the cell wall, plays a role in algal proliferation, and acts as a protective mechanism against biological factors [112]. According to Hidayati et al. [95], spirulina algae have a total of phenolic compounds that equate to 26.64 mg of gallic acid per gram of extract. Due to the redox characteristics that they possess, they play a significant role in antioxidant activity and are very efficient in scavenging harmful free radicals. The total quantity of phenols recorded in the spirulina extract was determined to be 2238.46 mg of gallic acid/kg of the extract, while the total amount of flavonoids found in the extract was determined to be 142.23 mg of quercetin/kg of extract [82]. Matos et al., [53] found that the alcoholic extract of spirulina algae (*A. platensis*), included the highest concentration of polyphenols. This concentration was 205 mg gallic acid/100 g dry weight, although the percentage was greater in the aqueous extract, which contained 334 mg gallic acid/100 g. According to the findings of Michael et al. [36], dry spirulina algae contained a significant quantity of total phenols, flavonoids, and carotenoids. The amount of phenols in spirulina extract was found to be 409.28 mg gallic acid/g, and the amount of flavonoids was found to be 13.25 mg rutin/g extract. Another study found that fresh and dried spirulina algae extract both contained a significant quantity of total flavonoids, with values of 22.10 mg/g and 10.91 mg/g, respectively [113]. According to the findings of Gabr et al. [114], the highest concentration of total phenols was found in spirulina biomass (51.20 µg/mL), then in the ethanolic extract (49.48 µg/mL), and the lowest concentration was found in the aqueous extract (15.26 µg/mL). When calculating the total flavonoids, it was observed that the amount of flavonoids acquired from algal biomass was the largest (97.73 μg/mL), followed by the amount received from the ethanolic extract (69.07 μg/mL), and the amount obtained from the aqueous extract was the lowest (4.67 μg/mL). When compared to algae and the extract made with ethanol, it was noted that the amount of phenolic and flavonoid chemicals present in the aqueous extract was significantly lower.

According to Salamatullah, [115], the methanolic extract of algae resulted in 3.46 mg/g of phenols, while the ethanolic extract resulted in 2.28 mg/g of phenols. The acetone extract of algae resulted in 7.37 mg/g of phenols, which was the highest value among the three extracts. In comparison to the other extracts, the quantity of total flavonoids that were produced by the methanolic extract, both with and without the addition of hydrochloric acid, was the greatest, reaching 6.37 and 6.05 mg/g, respectively. In contrast to the dried sample, fresh spirulina was shown to contain a greater number and higher quality of beneficial chemicals, according to the findings of another study. It was reported that the total flavonoids of fresh spirulina had 469.96 quercetin/g extracted in dry weight, whereas dried spirulina had 119.43 quercetin/g extracted in dry weight [116]. It may be deduced from the fact that the phenolic acids measured 38.64 and 7.50 mg gallic acid/g extract on a dry weight basis, respectively, that this is because of the variation in the amount of water present in the sample. Pyrogallol, gallic, chlorogenic caffeine, vanillic, p-coumaric, naringin, hespirdin, rutin, quercetrin, naringenin, catechin, and hespirtin are some of the phenolic chemicals that might be found in spirulina algae. Pyrogallol was found to contain the highest amount of phenolic compounds in both the biomass and the aqueous extract of spirulina, with a concentration of 638.50 and 12.33 mg/100 g, respectively. On the other hand, the compound E-vanillic acid contained the highest amount of phenolic compounds in the ethanolic extract, with 18.20 mg/100 g. Flavonoids such as catechein, epicatechein naringin, hespirdin, rutin, quercetrin, quercetin, naringenin, hespirtin, kampferol, and apigenin can be found in spirulina algae. In the spirulina biomass, the flavonoid component with the greatest concentration was hespirdin, which had a value of 9.013 mg/100 g. This was followed by ethanol and aqueous extract, which had concentrations of 0.652 and 0.359 mg/100 g, respectively [114].

The bioavailability of various phenols is affected differently depending on the type of food consumed [117]. Because it is found in enough quantities, spirulina may be regarded as a reliable source of phenolic compounds, In addition to being primarily found in fruits and beverages, such as tea, wine, and coffee, polyphenols can also be found in vegetables, leguminous plants, and grains [112]. The amount of polyphenols present in different foods can be attributed to a number of factors, including genetic, environmental, and industrial A variety of parameters, including the acidity function of the growth media, can have an effect on the total quantity of phenolic compounds that are present in spirulina. Abd El-Monem et al. [103] cultivated spirulina in a conventional growth medium by adjusting the pH values, and extracts of algae were made using a variety of solvents at a concentration of 70% (acetone, methanol, and ethanol). It was observed that the optimal pH was 10, which led to the maximum amount of flavonoids in the ethanol extract (7.6 mg/g) and the highest quantity of phenols in the acetone extract (0.52 mg/g) [103]. Therefore, they claimed that algae may produce phenols as a defense mechanism against disease, stress, depletion of nutrients, and an increase in the pH of the culture media. This resulted in an enhanced generation of phenols to reduce oxidative stress induced by high pH. Additionally, it was shown that the polyphenols in these algae include a greater quantity of total phenol compounds and a smaller quantity of total flavonoid compounds [36].

The determination of phenolic compounds in the alga *A. platensis* and their application in food preservation to increase the shelf life was the subject of just a few investigations. In order to achieve this goal, the treated product’s oxidative deterioration is decreased, and the product’s structural and organoleptic features are improved. The risk of developing illnesses that are brought on by oxidative stress is decreased by eating foods that are high in antioxidants. Even when utilizing a concentration that was greater than 40 mg/mL, it was determined that the extract of spirulina did not demonstrate any inhibitory effect against the stable radicals caused by the substance DPPH (1,1-diphenyl-2-picrylhydrazyl) [118]. In addition, Batista et al. [83] revealed that when estimating the amount of total phenols in biscuits enriched with spirulina algae, the addition of spirulina by 2% did not give any significant differences compared to the control sample, as the amount of phenols was 1.4 mg gallic acid/g, while the content of phenolics increased significantly to 1.6–1.7 mg gallic acid/g when spirulina was increased to 6%. El-Beltagi et al. [8] studied that the aqueous extract of spirulina contained a significant amount of total flavonoids, which was 79.6 mg/g dry weight, and that the amount of total phenols was 16.0 mg/g dry weight. Consequently, these were used to enhance pomegranate juice because of the positive effects that these chemical compounds have on one’s health. According to Martelli et al. [97], the presence of phenolic compounds in spirulina extract was associated with antimicrobial activity. Martelli et al. [97] also noted that the amount of phenolic compounds that showed the highest level of inhibitory activity against the bacteria that cause transmitted diseases was 3.18–3.40 mg gallic acid/g. This paves the way for the use of adding spirulina as a food preservative, as there are numerous phenolic chemicals that have the ability to inhibit the growth of microorganisms and are present in different kinds of algae.

## 4. Potential Health Benefits of Spirulina from a Human Nutrition, Biological, and Medicinal Standpoint

### 4.1. Spirulina for Formulation of Various Food Products in the Food Industries

#### 4.1.1. Snack Food Formulation

Because of its great nutritional value as a source of proteins and minerals, the biscuit and snack industry has been increasingly interested in incorporating algae, particularly spirulina, into its products. One option to increase and improve the nutritional content of this snack is to produce it with the addition of spirulina. This is one technique. The results revealed that the addition of spirulina at any of the following concentrations: 0%, 2.5%, 5%, 7.5%, 10%, or 12.5% led to an increase in the percentage of protein (9.43–18.11%) and ash (1.31–2.67%). After undergoing the microbiological testing, all of the samples were found to be risk-free, and the conclusions drawn from the sensory analysis pointed to the exclusion of the addition at the 12.5% concentration [2]. There is a growing trend in the manufacture of biscuits that involves the use of beneficial ingredients. Biscuits are a type of snack food that is consumed by a large number of people. Algae, such as spirulina and Chlorella, are used as a source of proteins, antioxidants, and biologically active molecules in the process of promoting wheat cookies. These algae are included among the additions. There were two different addition percentages, which were 2% and 6% by weight/weight respectively. The incorporation of 6% each of spirulina and Chlorella resulted in significantly increased protein content (13.2–13.5%) [83]. As a result, these additions are regarded as a source of protein, since the biscuit that was loaded with the spirulina alga *A. platensis* had the strongest antioxidant activity and received the highest score in the sensory evaluation [83].

The effect of adding spirulina to biscuits during the fortification process at varying rates of 0%, 5%, 10%, and 15% in comparison to regular biscuits has been researched in terms of its impact on the nutritional content of the biscuits [119]. Comparing the scores acquired by the control sample and the samples that had spirulina added to them, the findings of the sensory assessment revealed that the control sample had a lower score for the majority of the sensory qualities. Additionally, it was found that the incorporation of spirulina into biscuits resulted in an increase in both the percentage of protein (9.09 and 14.73%) and the amount of energy (554, 672 kCal), respectively, that they contained. The functional biscuits that are supplemented with spirulina algae are shown in Table 3A On the other hand, research carried out by El Nakib et al. [34] demonstrated that the nutritional content of biscuits might be improved by adding certain percentages of spirulina to the foods that schoolchildren eat for snacks. The percentages tested were 0%, 5%, 10%, and 15%. It also revealed an increase in fatty acids, such as omega-3, omega-6, omega-7, and omega-9, with omega-6 containing the highest percentage of unsaturated fatty acids, specifically linolenic and γ-linolenic acid, with values of 33.0 and 30.0 mg/g, respectively [3,83,84]. Spirulina is included in the formulation of the snacks and functional baby food listed in Table 3B.

#### 4.1.2. Pasta Formulation

Algae from the genus *Spirulina* have been put to use in the pasta-manufacturing industry in order to produce enriched pasta with enhanced nutritional, sensory, and therapeutic benefits [122]. It has developed a wide variety of new food products based on spirulina in order to cater to the requirements of the customers, such as sushi, jerky, and pasta [15]. Spirulina was added to wheat flour at a rate of 5% and 10% in order to prepare macaroni. As a result, the protein and energy content of the macaroni increased to 10.32% and 14.50%, respectively, while the caloric value increased to 322.94 and 327.60 kcal/g in the pasta that had been enriched with algae at a rate of 5% and 10%, respectively [124]. The chemical characteristics of the pasta were significantly improved as a result of the addition of spirulina at several percentages (0%, 0.25%, 0.5%, 0.75%, and 1% weight to weight), which resulted to a considerable improvement in the nutritional value of the pasta. In comparison to the sample that served as the control, the macaroni that had a concentration of 0.25% moss added to it received the highest score on the sensory evaluation. For this reason, this ratio was utilized throughout the production process of pasta in order to generate the greatest possible product that had been fortified in terms of its nutritional content, sensory value, and functional therapeutic capabilities [122]. In addition, Pagnussatt et al. [123] observed that the addition of spirulina and oats to dry pasta resulted in an increase in the product’s nutritive content, which was a result of the product being fortified. The amount of soluble solids included in the pasta as well as its color was impacted by spirulina, whereas the acidity value of the pasta was impacted by oats, which also enhanced the values of crude protein and overall food flavor (13.06%). These additions, when compared to the amount of fiber that is often found in commercial pasta (2.40%), can be regarded as a source of fiber, which lends them a healthful and nutritious value [123]. The functional pasta that has been enhanced with spirulina algae is shown in Table 3C.

#### 4.1.3. Formulation of Dairy Food Products

The production of a wide variety of dairy products includes the use of spirulina algae. Mocanu et al. [127] employed *S. platensis* to improve the nutritional content of fermented dairy products. During incubation and storage, the impact of *S. platensis* on the probiotics Bifidobacterium animalis ssp. lactis and Lactobacillus acidophilus was investigated. According to the findings, the consumption of spirulina over the course of the full storage time had a positive impact on the number of initiator bacteria B. animalis ssp. lactis and L. acidophilus that survived. Spirulina, which has a protein content of between about 55% and 70%, can be added to food products in order to increase the nutritional value of those products. When it came to the preparation of the soft-cheese product, the findings indicated that adding 1% of *S. platensis* was the optimal concentration from both the physicochemical and the sensory points of view. This addition had a considerable and favorable influence on the levels of protein, water, fat, and β-carotene, as well as on the tissues [116].

##### Ice cream

Because algae include various antioxidants, such as polycarotene phenols, which have the power to scavenge free radicals, the usage of algae in the making of ice cream has many positive impacts on one’s health. The ice cream product that was added to algae extract demonstrated an increase in antioxidant activity and gave the highest inhibition level of 39.7% in mint ice cream samples. This was indeed in comparison to the control sample, which did not include algae and resulted in an inhibition level of 32.8% [125]. In order to make a functional ice cream that had a high nutritional value, *S. platensis* was included in the production process. The optimum concentration of algae was 1.2%, which had a beneficial impact on the amount of protein in the product and had sensory and physical attributes that were satisfactory [116]. Although spirulina was employed as a stabilizer in the production of ice cream by Malik [40] with several replacement percentages (25, 50, 75, and 100%), they found that the optimal replacement percentage was 50% with a concentration of 0.15%. In comparison to the control sample, the treated product maintained the highest possible level of microbiological quality throughout the duration of the storage period. This treatment also had a favorable influence on the nutritional content of the product and improved its sensory characteristics. The spirulina-enriched functional ice cream is presented in Table 3D.

##### Yogurt and Acidophilic Milk

Functional yogurt was made using dried spirulina at different concentrations (0.1%, 0.2%, 0.3%, and 0.5%), with the concentration of 0.3% giving the best positive effect in enhancing the nutritional value and improving the sensory characteristics. In addition, it showed the highest survivability of S. thermophilus and L. bulgaricus compared with the control throughout the storage period [40]. Spirulina was also used in the preparation of yogurt at concentrations of 0.5% and 1% *w*/*w*, as it was discovered that adding 1% spirulina to yogurt had a positive effect on the numbers of lactic acid bacteria, which decreased in the control sample, while adding 0.5% was superior to adding 1% for the sensory characteristics of the product. Therefore, yoghurt that has been supplemented with spirulina is an excellent approach to keeping the viability of lactic acid bacteria for the thirty days that the product is stored in the refrigerator [130]. *S. platensis* was added to milk that had been fermented by probiotic bacteria using a starter (ABT-4) that contained L. acidophilus (A), Bifidobacteria (B), and Streptococcus thermophilus (T). The effect of this addition was evaluated. According to the findings, including *S. platensis* in the mix had a beneficial impact on increasing the number of initiator bacteria that made it through the storage period [134,135]. When making soy yogurt that was enriched with spirulina, it was observed that the optimal addition ratio was 0.80% (*w*/*w*), which produced the ideal yogurt at a temperature of 40 °C and a fermentation time of 12 h. This was found when the yogurt was being prepared [131]. The dairy products that have been fortified with spirulina are listed in Table 3E.

### 4.2. Biological and Therapeutic Significance of Spirulina

**Figure 2:**
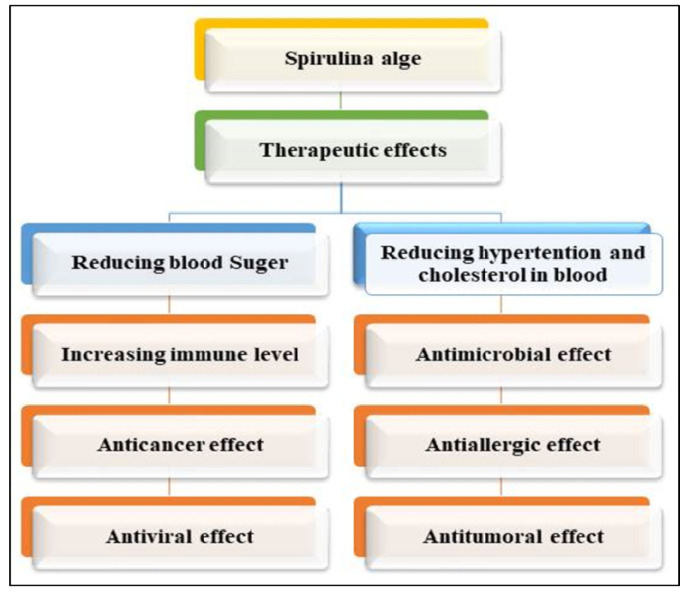
A diagrammatic explanation of the therapeutic value conferred by spirulina algae.

Spirulina, especially *A. platensis*, has a high nutritional value, does not harm the environment, and possesses remarkable curative capabilities. According to studies, spirulina plays a role in the treatment of a number of diseases, and it can be utilized in the reduction of blood sugar levels, the reduction of blood pressure, the modification of dysbiosis caused by an unbalanced intestinal flora, anticancer, antioxidant, antibacterial, anti-allergic, antiaging, and anti-inflammatory activities, as well as in the reduction of HIV immunity [112]. The chemical composition of spirulina, which includes minerals (especially Fe), phenols, phycocyanin, and polysaccharides, is primarily responsible for its beneficial effects on human health. The clinical data that are now available do not show that ingesting spirulina poses a threat to one’s health, as stated by the US Food Supplements Convention [45]. The World Health Organization (WHO) verified that not only is spirulina a good diet due to its high Fe and protein content, but it may also be given to children without any adverse effects [39]. Figure 2 demonstrates the biological as well as therapeutic benefits of the spirulina algae. In addition to that, it has been described in further detail in Table 4.

#### 4.2.1. Importance in Weight Control

Several different studies have pointed to the relevance of spirulina in maintaining a healthy weight. Spirulina may help regulate body weight by inhibiting the migration of macrophages into visceral fat, preventing the buildup of fat in the liver, lowering the levels of oxidative stress in the body, and increasing insulin sensitivity and satiety. These are the hypothesized mechanisms of action. In their study, DiNicolantonio et al. [155] validated the effects of spirulina on weight loss. They found that obese adults who consumed 1–2 g of spirulina per day for a period of three months saw a reduction in their body weight, body mass index (BMI), and waist circumference. A considerable rise in body weight, total protein, albumin, and hemoglobin levels were seen in diabetic rats after they were given an aqueous extract of spirulina to consume for a period of fifty days. This points to an improvement in general health conditions as well as metabolic mechanisms brought about by efficient glycemic management. On the other hand, the weight of diabetic animals that were not treated dropped [55].

**Figure 3:**
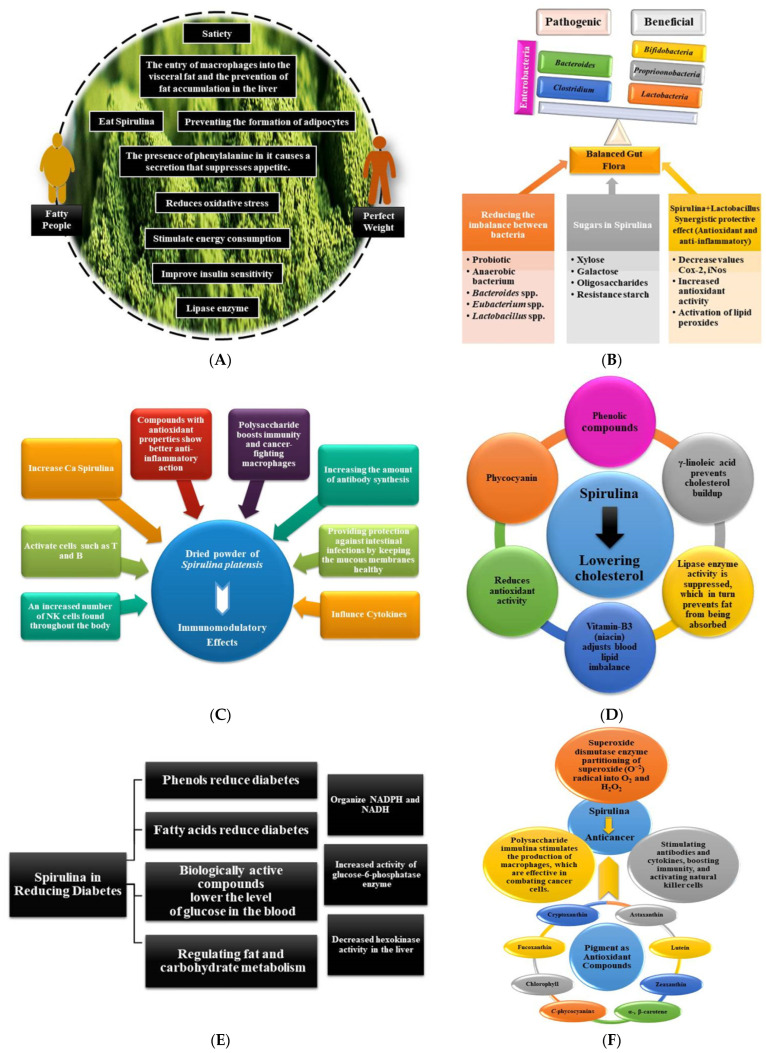
A pictorial representation of the numerous beneficial health effects that have been derived from consuming spirulina algae and their associated bioactive components. (**A**) Role of spirulina algae play in the process of weight loss; (**B**) How consuming spirulina algae might alter the pattern of the intestinal balance; (**C**) Role played by spirulina algae in the regulation of immunity; (**D**) Function that spirulina algae play in reducing cholesterol levels; (**E**) Role that spirulina algae play in lowering the risk of developing diabetes; and (**F**) Anticancer properties of the spirulina algae.

According to the findings of the research conducted by Huang et al. [156], consuming spirulina supplements in doses ranging from 1 to 19 g per day for a period of time ranging from 2 to 48 weeks had a beneficial impact on a variety of key indicators of the body. It was noticed that there had been an increase in overall body weight as well as in BMI. When compared to the rats who were fed the control diet, the rats that were given either pomegranate juice, spirulina extract, or both together had a considerable rise in their body weight. These liquids stimulated hunger, which ultimately resulted in weight gain [8]. In addition, Moradi et al. [157] found that supplementation with spirulina significantly lowered body weight from −1.98 to −1.14 kg, notably in obese adults at a dosage of 4 g/day from −2.45 to −1.68 kg. These results were found when the participants were given spirulina. The decline was larger than the people who were overweight (−1.62 to −0.93 kg), the percentage of body fat reduced from −1.02 to −0.54 kg, and the waist circumference decreased from −1.40 to −1.39 cm. It has been observed that spirulina has beneficial effects on weight and waist circumference when used for at least 12 weeks, and it has a positive effect on BMI when used for a longer period of time. Chronic diseases are associated with obesity and overweight, and it has been observed that spirulina has these beneficial effects [158]. Supplements containing spirulina contain antioxidants, which are known to have a significant role in the management of weight for diabetics and those who are obese. They increase the body’s need for energy while simultaneously preventing the production of adipocytes and the enzyme lipase. The usage of these supplements resulted in a substantial reduction in body weight from −1.76 to −0.88 kg and a significant reduction in waist circumference from −1.40 to −1.39 cm; however, there was no significant effect on BMI observed. Spirulina was found to reduce lipid accumulation in the liver by inhibiting macrophage infiltration into visceral fat. Additionally, the phenylalanine content of spirulina was found to be reduced, which may have led to an increase in the secretion of cholecystokinin, a hormone that suppresses appetite [159]. Figure 3A is a diagrammatic illustration of the function that spirulina algae play in the process of weight reduction.

#### 4.2.2. Importance in Intestinal Flora

The findings of studies on spirulina suggest that it plays a role in the modulation of dysbiosis, which is an imbalance in the intestinal flora. This suggests that the combination of spirulina and probiotics could represent a new strategy for improving the growth of beneficial intestinal microorganisms [150,160]. The aqueous extract of S. platensis was shown to be the greatest source of the biostimulators xylose, galactose, oligosaccharides, and resistant starch. These biostimulators had a tremendous effect on boosting the development of probiotic bacteria. The biostimulators encouraged the growth of beneficial bacteria in the colon, such as lactic acid bacteria, Bifidobacteria, and Lactobacilli, while also reducing the number of dangerous bacteria, such as Enterobacteria and Clostroids [161]. Spirulina has been shown to have a positive impact on human and animal health by altering the composition of gut bacteria and boosting the growth of useful bacteria. This has the knock-on effect of enhancing overall health. Changes in the composition of the bacteria in the intestinal tract are a contributing factor in the development of a wide variety of disorders, including those affecting immunity and metabolism. There are a wide variety of disorders that are linked to microorganisms, one of which is inflammatory bowel disease (IBD). It has been observed that consuming spirulina brings about a reduction in the imbalance that exists between the genera of beneficial probiotic bacteria and the genera of natural anaerobic bacteria, particularly *Bacteroides* spp., *Eubacterium* spp., and *Lactobacillus* spp. [150,162,163,164]. According to the findings of research that was conducted by Abdel-Moneim et al. [165], alcoholic spirulina extracts (methanol, acetone, and hexane) and biosynthesized Se nanoparticles (SeNPs) both possessed significant antioxidant activity and antibacterial activity. The pathogens included three strains of Gram-positive bacteria, three strains of Gram-negative bacteria, and three strains of *Candida* spp. and *Aspergillus* spp. The spirulina methanolic extract had the highest antibacterial and antioxidant activity levels, as well as the highest total phenol content. The total phenol content of spirulina and SeNPs, as well as their biological activity, were found to have a correlation with one another. In the realm of the effectiveness of antibacterial and antioxidant agents, with the potential for safe medicinal uses as alternatives to traditional chemical medications and antibiotics. The capacity of functional foods to change the composition of the gut microbiota is the source of their many health advantages. These foods also have the potential to interfere with patients’ existing health conditions [166]. It was observed that the antioxidant and anti-inflammatory actions of spirulina and Lactobacillus bacteria had a complementary impact on the treatment of ulcerative colitis (UC). When the mice were given Lactobacillus at a rate of 1 × 10^9^ CFU per mouse per day and spirulina at a rate of 500 mg/kg per day, the standard effect of both was determined. It has been demonstrated that the protective benefits are caused by its capacity to lower levels of the inflammatory markers iNOS and COX-2, as well as boost antioxidant activity and significantly prevent lipid peroxidation [167]. Figure 3B is a diagrammatic description of how ingesting spirulina algae may affect the pattern of the balance in the digestive tract.

#### 4.2.3. Immunological Importance

In terms of the immune system, spirulina has a major role to play in boosting immunity due to the fact that it possesses a wide range of biological activities and is important to nutrition due to the high concentration of natural nutrients it contains [117]. In addition, it can modulate the immune system and the bioactivity of macrophages by activating T and B cells, promoting the production of antibodies and cytokines, increasing the concentration of NK cells in tissues, and encouraging the generation of antibodies [84]. Spirulina is a rich source of antioxidants and anti-inflammatory substances, which are able to not only support and develop physiological functions of the nervous system and brain, as well as compensate for nutritional deficiencies, but also promote a beneficial immune response [5]. Several studies have also demonstrated the positive effects of spirulina, including the immunological and antioxidant effects that are attributable to its carbohydrate content, particularly polysaccharides. These effects are due to the fact that spirulina has a high concentration of polysaccharides [44,100].

The chemical composition of spirulina (*A. platensis*), which is rich in minerals and carbohydrates and consists of polymers including glucose and branching polysaccharides comparable to glycogen, is largely responsible for the positive effects on human health that these microalgae have. These high molecular weight compounds of negatively charged sugars are referred to as “Immulina,” and they have significance in the pharmaceutical industry. It was discovered that after ingestion at a rate of 400 mg per day for seven days, they enhanced the activity of natural degrading cells against cancer cells. This finding gives them importance in the pharmaceutical industry. In addition to this, the antiviral activity of these drugs has been demonstrated against types I and II of the herpes simplex virus [6,43,168,169]. In most cases, this immunoreactivity is brought on by Ca spirulan, which is made up of the following saccharides: rhamnose, methylrhamnose (acofriose)-3-O, methylrhamnose di-O-2,3, methylxylose-3-O, uronic acids and sulfates [27,33,45,170,171].

Spirulina is utilized to produce phycobiliproteins, which are then put to use as colorants. According to the findings of a large number of studies, these pigments offer a wide range of potential uses. In clinical medicine and immunological analysis, as well as for therapeutic and diagnostic reasons, they are utilized as fluorescent material. It was also revealed that it had a considerable effect on lowering the quantity of cholesterol detected in the blood and that it acted as a protective agent against hepatitis [120,172,173].

Spirulina is a stimulant of immune system cells because it increases anti-inflammatory resistance, stimulates the synthesis of antibodies and cytokines, activates macrophages, stimulates natural killer cells, and activates T and B cells. Consuming spirulina helps to keep the intestinal epithelium, which is the first line of defense of the mucosal barrier against infections, in good working order [44,89]. Figure 3C presents a diagrammatic description of the role that spirulina algae play in the control of immunity.

#### 4.2.4. Blood Pressure Treatment

It was observed that supplementation with spirulina led to a substantial drop in diastolic blood pressure (DBP), but at the same time, it led to a rise in systolic blood pressure, which is one of the indications of cardiovascular safety and metabolism in humans [156]. This finding suggests that spirulina has a beneficial influence on blood pressure. Vasodilation is the mechanism that has been demonstrated to be responsible for spirulina’s ability to alleviate hypertension in mice [94]. When taken orally at a dose of 4.5 g per day for a period of six weeks, spirulina is related to a drop in both systolic and diastolic blood pressure. Because it contains the pigment C-phycocyanin, it was also reported to have a lipid-lowering effect. The high K and relatively low Na content of spirulina have beneficial effects on blood pressure. It was shown that C-phycocyanin stops platelets from adhering to one another by preventing Ca mobilization and moderating free radicals that are generated by platelets. Spirulina also helps prevent atherosclerosis [89]. Lipoproteins are not only used as a coloring agent but they have also been associated with a wide variety of beneficial impacts on one’s health [99]. When rats with naturally high blood pressure were given phycocyanin pigment in their regular diet at doses of 2500, 5000, or 10,000 mg/kg over a period of 26 weeks, hypotension was found. The ACE-I inhibitor peptide, which has been shown to have an effect on lowering blood pressure, was discovered during the process of phycocyanin synthesis [174].

#### 4.2.5. Cholesterol Treatment

Spirulina seems to have a positive effect on metabolic risk factors, particularly elevated blood lipid, according to a number of studies. It was noted that spirulina has an effect on lowering blood lipids, particularly triglycerides and cholesterol linked to low-density lipoprotein (LDL), as well as an indirect effect on total cholesterol and cholesterol linked to high-density lipoprotein (HDL), due to the presence of a pigment called phycocyanin [89]. Phenolic compounds are antioxidants that protect cells and natural chemicals in the body from the damage caused by free radicals. Free radicals are responsible for causing tissue damage in the body as well as oxidizing LDL cholesterol, which can lead to heart disease if it accumulates in the arteries [6]. Consuming spirulina can help lower levels of dangerous LDL and triglycerides, and it can also play a part in the indirect modification of high cholesterol and total cholesterol levels. This is because the components of spirulina have antioxidant action, and the aqueous extract of spirulina reduces the absorption of fats from foods by reducing the activity of the lipase enzyme that is released by the pancreas [94]. This is why spirulina is so beneficial. When investigating the impact of the ethanolic extract of spirulina on lipid contents in the blood of artificially hypercholesterolemic male laboratory rabbits at two different dosages (33 and 66 mg/kg) over the course of four weeks, the researchers found that the extract had no effect [175]. It was revealed that there was a reduction in the levels of cholesterol, triglycerides, LDL, and dangerous LDL, while there was an increase in the levels of HDL, which was favorable.

Over one month, hypercholesterolemic rabbits had their total cholesterol, triglycerides, and HDL levels monitored to determine how they were affected by a diet that included 0.5 g of spirulina per day as a dietary supplement [176]. An increase in the level of HDL in the blood was shown to be associated with a decrease in the levels of total cholesterol found in the blood, but there was no discernible change in the levels of triglycerides found in the blood. According to the findings of the study, this is because spirulina contains natural antioxidants such as phenolic compounds, γ-linolenic acid, and phycocyanin, all of which work to prevent hypercholesterolemia and the negative consequences of the condition. Spirulina algae, when consumed as a functional food, has been shown to aid in the treatment of cardiovascular disorders such as high cholesterol and atherosclerosis, as validated by Wang et al. [162]. In addition to this, it regulates the level of LDL and HDL in the blood, which results in favorable effects on high blood lipids. It was noted that taking spirulina supplements had a beneficial impact on blood lipid parameters, as it was observed that the values of triglycerides, total cholesterol, LDL, and very LDL decreased, while the value of HDL increased. This was found to be the case because it was found that the values of HDL increased [156]. Spirulina can contribute to in avoiding the production of cholesterol, which is mediated by γ-linolenic acid. A lack of γ-linolenic acid can cause the artery wall to become thicker, which can lead to high blood pressure and an increase in blood lipids. In addition, spirulina includes vitamin B3 known as niacin, which corrects an imbalance in the lipids of the blood [155]. Figure 3D provides a graphic of the role that spirulina algae play in lowering cholesterol levels.

#### 4.2.6. Diabetes Treatment

One of the metabolic illnesses, diabetes is also one of the most common diseases and a major cause for concern all over the world owing to the toll it takes on public health. Diabetes is a metabolic ailment [117]. Spirulina demonstrated both glucose and lipid modulation activities, indicating that it may have a regulatory function in the metabolism of carbohydrates and lipids in both experimental animals and diabetics [84]. Gheda et al. [55] found the presence of a variety of bioactive compounds when they conducted an analysis of the methanolic extract of S. platensis using GC-MS. These bioactive compounds included phytols, phenolic compounds, and methyl esters of fatty acids. These bioactive compounds worked together to produce a synergistic effect. These are what give spirulina its antioxidant effects, as well as its ability to decrease cholesterol and blood sugar levels. In addition, it was realized that administering the methanolic extract to experimental mice at doses of 15 and 10 mg/kg body weight resulted in hypoglycemic activity and improved the histological disorders of the liver and pancreas that are associated with diabetes. This was found to be the case when the mice were given the extract. According to the findings of the study, this algae should be included in the formulation of medicinal products intended for the treatment of diabetes and the symptoms associated with it [55].

During the in vivo phase of the research, diabetic mice were given an extract of spirulina to cure their condition. The methanolic extract of spirulina caused hypoglycemic activity when it was administered at concentrations of 15 and 10 mg/kg by body weight. This activity was characterized by a reduction in a variety of liver and kidney functions, an increase in fats, and an improvement in the histological disorders of the liver and pancreas that are associated with diabetes [55]. The presence of these chemical molecules with biological activity, which may have the potential to serve as antioxidants in a synergistic way and may also have an impact that is diabetic-lowering. Spirulina has also been demonstrated to be useful in decreasing the level of glucose that is present in the blood when the individual is fasting [94]. Diabetic people who consume spirulina have lower blood sugar levels and are less likely to experience problems. Spirulina also controls cholesterol levels. Due to the fact that spirulina possesses antioxidant and immunomodulatory qualities, diabetic individuals might benefit from consuming it as a functional food in order to better manage their type 2 diabetes. Spirulina supplementation can be used to maintain nutritional balance, reduce blood lipids and modify carbohydrates, and drop blood sugar levels by a large amount. It can also be used to minimize inflammatory stress levels [44,156].

The majority of spirulina’s effect can be attributed to the upregulation of NADPH and NADH. NADH is a cofactor in lipid metabolism that is responsible for the high activity of glucose-6 hydrogen ion phosphatase (H+). This enzyme binds to NADP+ in the form of NADPH. Spirulina’s effect can also be attributed to the high activity of glucose-6 hydrogen ion phosphatase (H+). This help is necessary for the synthesis of lipids from carbohydrates, and spirulina may be able to oxidize NADPH. Spirulina is a blue-green alga. One animal investigation revealed that the activity of hexokinase in the liver of diabetic control mice was greatly reduced, whilst the activity of glucose-6-phosphatase was significantly enhanced. Both of these changes were observed in the liver. The administration of spirulina to diabetic mice led to an increase in hexokinase activity and a reduction in glucose-6-phosphatase activity. Mice that were given spirulina had higher levels of hexokinase activity, which showed that the mice’s hepatocytes took in more glucose from the blood [89]. Figure 3E presents a schematic representation of the function that spirulina algae perform in reducing the likelihood of an individual getting diabetes in the future.

#### 4.2.7. Cancer Treatment

There is a tremendous opportunity for the potential new natural anticancer compounds from BGA, particularly spirulina, in the treatment of cancer. It has been demonstrated that the high concentration of antioxidant molecules found in algae is what makes it so effective as an alternative treatment for a variety of diseases, including cancer, diabetes, and inflammation [3,114]. Spirulina has been shown to have therapeutic effects, such as protection against some types of cancer, enhancement of the immune system, protection against radiation, and reduction of high blood fats and obesity, according to several studies. Figure 3F presents a schematic representation of the anticancer effects that may be attributed to the consumption of spirulina algae. It was reported by Hosseini et al. [33] that the spirulina algae product possesses many pharmacological activities, such as anticancer, antiviral, antimicrobial, anti–heavy metal, immunomodulatory, and antioxidant due to its high content of protein and PUFAs, particularly the high content of γ-linolenic acid. These pharmacological activities are attributed to the high content of γ-linolenic acid [45]. Spirulina has been shown to boost the stimulation of antibody and cytokine production, as well as NK activation. Additionally, it has the capacity to augment human immunity and plays a role in inhibiting the growth of tumors. Spirulina exerts its effects on human myeloid progenitors and natural killer cells in either a direct or indirect manner, depending on the context [94].

The astaxanthin tincture has potent anti-inflammatory and antioxidant capabilities, which have the capacity to prevent or lessen the severity of a wide variety of ailments, including cancer. Lutein, zeaxanthin, and β-carotene are known to reduce the risk of developing premenopausal breast cancer. Cryptoxanthin and α-carotene are known to reduce the risk of developing cervical cancer. Chlorophyll tincture has been demonstrated to have both antioxidant and anticarcinogenic action, while fucoxanthin pigment has been proven to have antiobesity and anticarcinogenic effects. As a result of this, the food industry has begun to make use of spirulina pigments as a coloring ingredient [98,177,178].

At a concentration of 50 μM for up to 48 h, C-phycocyanin and β-carotene had an anticancer impact on the human chronic myeloid leukemia (K562) cell line, lowering 49% of cell growth [44]. This feature of spirulina can be related to the presence of antioxidants with a high level of β-carotene and superoxide dismutase enzyme in the food. Spirulina has been shown to have a protective impact against cancer cells [89]. Spirulina extract was tested at five different concentrations to see whether or not it has an anticancer effect: 6.25, 12.5, 25.50, and 100 μg/mL. The cytotoxicity test revealed that cells were inhibited by 50% at a concentration of 19.18 μg/mL after 72 h. The results demonstrated that the extract had a highly toxic impact against cancer cells, and this effect increased with increasing concentration [179].

The cytotoxicity of spirulina extract was demonstrated against cancer cell lines when it was tested on colon cancer (HCT-116), liver cancer (HepG2), and CACO colon cancer cell lines. The LC50 values for each of these cancer cell lines were 21.8, 14, and 11.3 mcg/mL, respectively. This effect was attributed to the presence of antioxidant plant pigments (carotenoids, chlorophyll, and phycocyanin), as well as the presence of polysaccharides, as the use of spirulina extract has been suggested in the development of anticancer drugs. Phycocyanin is a pigment found in blue-green algae [8]. When employing algal extract at a concentration of 1000 µg/mL, the findings of Matos et al. [53] indicated that spirulina was cytotoxic to Hela cells by 61.4% and reduced cell viability by 39%. This was the case when the cells were exposed to spirulina.

#### 4.2.8. Cardiovascular Disease Treatment

Cardiovascular disease and other circulatory disorders are the main causes of death and illness on a global scale [3,117]. The World Health Organization (WHO) says that cardiovascular diseases (CVDs) are responsible for the deaths of 17.3 million people a year, and it is anticipated that this figure would increase to more than 23.6 million by the year 2030. The primary contributors to CVDs are the formation of lipids in the blood and prolonged exposure to high levels of stress [156]. Spirulina, which is classified as a BGA, can be consumed either as a whole meal or as a dietary supplement. It is commonly taken in many Asian countries, where the incidence of several ailments that are prevalent in Western society, such as coronary heart disease, cancer, and arthritis, is far lower than in those countries [115].

According to a number of studies, taking spirulina supplements may help reduce the risk of CVDs and other conditions linked to atherosclerosis. It was shown that it plays a function in lowering triglycerides, total cholesterol, and LDL cholesterol while simultaneously raising HDL cholesterol levels. The consumption of spirulina by the experimental animals helped to keep the level of lipids in their blood unchanged to a substantial degree. Because of these features, which are a result of the presence of phycocyanin molecules, phenolic compounds, and PUFAs, spirulina was regarded to be a functional food capable of reducing cholesterol levels and avoiding atherosclerosis [180]. Patients who suffer from both high blood pressure and high blood lipids have an increased chance of experiencing a heart attack. It is necessary to bring LDL and DBP levels, as well as weight and blood sugar, down to normal levels in order to both prevent and cure this condition. Because of the significant antioxidant activity that spirulina supplements possess in human beings, their consumption holds a great deal of promise for the treatment and prevention of CVDs. It has been demonstrated that using these supplements has a beneficial impact on a number of metabolic and cardiovascular health markers in people, including triglycerides, total cholesterol, LDL, extremely LDL, fasting blood glucose, and blood pressure, without causing any negative side effects [156].

Consumption of PUFAs is linked to a reduced risk of death from a variety of illnesses, including cardiovascular disease and other conditions. EPA (20:5 n3) and DHA (22:6 n3) are two of the PUFAs that can be found in the spirulina [53]. The pigment phycocyanin in spirulina, which has properties comparable to those of the pigment bilirubin in terms of its structure, is thought to be responsible for the preventive effect that spirulina has against CVDs. It has been proved that the pigment bilirubin, which is present in bile, contains powerful antioxidants. These antioxidants prevent oxidative stress and the production of radical byproducts in plasma proteins and aromatic amino acid residues [162].

#### 4.2.9. Other Disease Treatment

Spirulina has been demonstrated to have a beneficial impact on the activation of glial cells as well as the treatment of neurodegenerative disorders. These conditions manifest themselves as a result of a deficiency in the body’s naturally occurring antioxidants and anti-inflammatory defensive mechanisms. This makes the brain more susceptible to the damaging effects of free radicals, such as ROS and RNS, which play an important role in the majority of neurological disorders, such as Alzheimer’s disease, Parkinson’s disease, multiple sclerosis, inflammatory lesions, and aging [5]. Piovan et al. [181] found that spirulina extract, which blocks lipopolysaccharides, helped control the activation of microglia and prevent the occurrence of neuroinflammation when they studied the neuroprotective effects before and after early treatment with the extract. This was the case when they compared the effects before and after early treatment with the extract. This is because there are a large number of bioactive chemicals present. Chlorophyll, pheophytin, carotenoids, β-carotene, and zeaxanthin are the components that have a positive impact on one’s health. In addition, Abdullahi et al. [182] pointed out the neuroprotective role of spirulina in mitigating the effects of spinal cord injury and its protective ability for the spinal cortical tracts and behavioral recovery in laboratory injured rat models. It was noticed that the optimal concentration of spirulina for rats was 180 mg/kg, and this concentration was found to be the most effective for rats [182].

Linolenic acid has several health benefits, including the ability to develop and maintain healthy bones, alleviate back pain, prevent arthritis and osteoporosis, avoid kidney stones, and protect teeth by ensuring that jaw bones remain strong. The benefits of γ-linolenic acid include enhanced brain function, alleviation of osteoporosis symptoms, enhancement of insulin metabolism, facilitation of the elimination of kidney stones, promotion of metabolic processes, defense against oxidative stress, and prevention of vitamin D shortage [183,184,185,186].

Some disorders that are linked to food may be traced back to a lack of certain nutrients or an inability to absorb them, including PUFAs. Being a rich source of γ-linolenic acid, in addition to linoleic and oleic acids, γ-linolenic acid is the most vital of the three. This particular acid makes for around 20% of the overall PUFA content that can be found in spirulina. The fact that unsaturated fatty acids have a beneficial effect on the majority of the chronic diseases plaguing modern civilization, such as cancer, diabetes, heart disease, arthritis, and Alzheimer’s disease, is one of the primary reasons for their significance. Because of the influence that they have on prostaglandins and leukotrienes, they are essential for the proper functioning of the cardiovascular system as well as the immunological system. As a result, the food and pharmaceutical industries have begun using these algae as a complementary food in order to treat a variety of health conditions [31,43,48,79,187,188].

Chlorophyll, carotenoids, and phycobilins are the three pigments in algae that are considered to be the most significant. These pigments have been shown to have a beneficial impact on a variety of health and therapeutic uses [2,189,190]. Spirulina has several benefits and is essential for the development of young children. It is also an excellent choice for adolescents and adults who are still developing their bodies. Because it is high in Ca and Fe and contains a little amount of selenium, spirulina is beneficial in situations of general weakness and poverty. It also protects against osteoporosis, and blood illnesses, as its Ca, Fe, and Se concentrations are, respectively, 1043.62, 338.76, and 0.0488 mg/100 g.

## 5. Concluding Remarks

Spirulina is the cyanobacterium species that has received the most interest from researchers in the pharmaceutical and food sectors. Researchers, analysts, and scientists from all around the world have conducted numerous studies and research projects on rising trends and new technological breakthroughs. As a result of this, a number of papers have been published during the last several years. As a consequence, we wished to gather all of the scattered material and consolidate it into a single source that will give crucial information to researchers and scientists in their ongoing research, scientific pursuits, and industrial initiatives in the future. As an outcome of the facts offered in this article, we can conclude that consuming spirulina algae has the potential for monetary gain as well as health advantages. This is mostly due to the contents of these algae, as well as their capacity to synthesize a wide range of chemical compounds that are both biologically and commercially important. As a natural byproduct, a substantial amount of biomass may be produced at a low cost for food processing, with the certainty of getting naturally occurring components with high nutritional value. Polyunsaturated fatty acids, carotenoids, phycobilins, polysaccharides, vitamins, sterols, and a wide range of bioactive compounds, such as antioxidants and cholesterol reducers, are among the naturally occurring substances that can be utilized in the development of functional foods. Despite the fact that the nutritional, environmental, and social advantages of spirulina have been gathered from a variety of published literature, it can be extrapolated that spirulina production is still limited to a few natural regions. As a result, a growing number of researchers and scientists worldwide are advocating for the wider production of spirulina.

## Figures and Tables

**Table 2:** A comparison of the relative protein content of spirulina algae with other food and food products based on the literature.

Food and Food Products		RPC ^a^ (%)	References
Spirulina		55.70	[59]
		55–70	[23]
		30–55	[60]
Beef		20.71	
Chicken		21.96	[61]
		22.25%	[62]
Fish	Carp	16.70	[63]
	Cod	17.40	
	Herring	18.10	
	Salmon	18.40	
Whole egg		12.60	[64]
Sausage		14.43	[65]
Milk	Buffalo	4.17	[66]
	Camel	3.38	
	Cow	3.56	
	Goat	3.44	
	Sheep	4.35	
Whey Protein	Buffalo	0.72	
	Camel	0.58	
	Cow	0.53	
	Goat	0.54	
	Sheep	0.74	
Whey Proteins		54.8 3.0–74.8 4.1%	[67]
Whey Protein Concentrate Powder		33.30	[68]
White Cheese		16.20	[69]
Organic hard cheese		21.53–25.70	[70]
Soy bean		38.30–40.30	[71]
		35.35–39.80	[72]
Common Oat		11.61	[73]
Oat grains		9.70	[74]
Black Bean (Organically produced)		25.20	[75]
Maize		12.65–12.45	[76]
Rice		7.76–8.31	[77]
Wheat		11.88	[78]

^a^ Relative Protein Content.

**Table 3:** Various developed functional food products with incorporation of spirulina biomass.

Type of Functional Food Products	*Spirulina* Biomass	Other Ingredients	References
**(A) Functional Biscuits**			
Cookies	10–15%	–	[34]
	5%, 10%, and 15%	–	[119]
Wheat Crackers	2% and 6%		[83]
**(B) Functional Snacks and Baby Food**			
Snack	0%, 2.5%, 5%, 7.5%, 10%, and 12.5%	–	[2]
Snakes for the elderly	750 mg/100 g	–	[120]
Baby food formulation	0%, 2.5%, 5%, and 7.5%	–	[39]
**(C) Functional Pasta**			
Pasta	1–15	–	[121]
	0%, 0.25%, 0.5%, 0.75%, and 1%	–	[122]
	0%, 0.6%, 2.0%, 3.4%, and 4.0%	–	[123]
	0%, 0.25%, 0.5%, and 0.75%	–	[124]
Novel Food Products Pasta, Maki-Sushi, Jerky Pasta,	Spirulina filled	–	[15]
**(D) Functional Ice Cream**			
Ice cream	5%	–	[125]
	0.15%	Fructo oligosaccharides (FOS) and Probiotic Vegetable Milk	[126]
	0%, 0.6%, and 1.2%	–	[116]
	0.075%, 0.15%, 0.23%, and 0.3%	–	[40]
**(E) Functional Dairy Products**			
Ayran Yoghurt	0%, 0.25%, 0.5%, and 1%	*Bifidobacterium lactis*, *Lactobacillus acidophilus, L. bulgaricus* and *Streptococcus thermophilus*	[127]
Low-fat Probiotic Yogurt	0.1–1%	Algae: *Spirulina platensis* and *Fexulago angulata*	[128]
		Bacteria: *L. delbruckii* subsp. *bulgaricus*, *L. acidophilus* ATCC 4356, and *S. thermophiles*	
Probiotic Fermented Milk Product	–	–	[127]
Soft cheese	0%, 1%, and 1.5%	–	[116]
Yoghurt	0%, 0.5%, 0.75%, 1%, 2%, and 3%	*L. acidophilus, L. delbruckii* subsp. *bulgaricus* and *S. thermophilus*	
	0%, 0.5%, and 1%	*Bifidobacterium animalis* ssp. *Lactis* (BB-12) and *L acidophilus* (ha-5)	[129]
	0.3%, 0.5%, and 0.8%	*L. acidophilus*, *L. delbruckii subsp*. *bulgaricus, S. thermophiles,* and spinach (10–13 *w*/*w*%)	
	0.5% and 1%	*L. acidophilus, L. delbruckii* subsp. *bulgaricus* and *S. thermophilus*	[130]
Soy Yoghurt	0.80%	*L. delbruckii* subsp. *Bulgaricus* and *S. thermophilus*	[131]
Functional Yogurt	0.1%, 0.2%, 0.3%, and 0.5%	–	[40]
Probiotic Fermented Milks	–	–	[132]
Fermented Symbiotic Lassi	0%, 0.05%, 0.1%, 0.2%, 0.3%, 0.4%, and 0.5%	*L. helveticus* MTCC 5463 (V3) and *Streptococcus thermophilus* MTCC 5460 (MD2)	[133]
Acidophilus Milk	0.5% and 1%	*L. acidophilus*	[130]
Acidophilus bifidus- thermophiles Fermented (ABT) Milk	3 g/L	*Bifidobacteria, L. acidophilus,* and *S. thermophiles*	[134]

– Not reported.

**Table 4:** Biofunctional effect of spirulina algae biomass application.

Biomass of Spirulina and Associated Products	Remarkable Observations		References
	Nutritional and Biochemical Component	Health and Other Beneficial Remarks	
Powdered/Dried solution 10%	Phenolic compound (phycocyanins, β-carotene), fatty acid composition (PUFAs), polysaccharide, vitamins, and mineral	Antioxidant properties	[130,136]
Spirulina extracts 70% (acetone, methanol and ethanol)	Phenolic and flavonoid compounds	Antimicrobial activity	[103,137]
10%, 20%, 30%, 40%, and 50%	Polysaccharides, phenolic compounds, phytopigments (carotenoids, chlorophyll phycocyanin) and phycocyanobilin	Anticancer and antioxidant properties	[8]
–	Phycocyanin	Anticancer, antidiabetic, and anti-inflammatory properties	[138]
–	Polyphenols and phycocyanin	Antioxidant and anti-inflammatory properties	[139]
–	Polysaccharides, protein, polyunsaturated fatty acid, vitamins, minerals, phenolic, pigments, sterols, and volatile compound	Antioxidant, anticancer, anti-inflammatory, antidepressing, antihypertensive, antiaging activities as well as arthritis, cardiovascular effusions, hypertension, increasing HDL cholesterol and reducing triglyceride properties	[140,141]
–	Protein, amino acid, unsaturated fatty acids, vitamins (A, B2, B6, B8, B12, E, and K), minerals (Fe and Ca) and antioxidant compound	Antiinflammatory, antitumoral, antivirial activities as well as properties of reducing blood lipid profile, blood sugar, body weight, and wound healing time	[142,143]
–	Protein, essential fatty acids (omega-3 and omega-6 type), vitamins (A, B1, B2, B12, and B3), minerals (Cu, Fe, Mg, Mn, and K), and pigments (xanthophyll and carotenoids)	Decrease LDL cholesterol, triglyceride levels, blood pressure, and blood sugar, while, increase hemoglobin level of red blood cells. Furthermore, it improved immune system	[144,145,146,147,148,149]
–	Polysaccharides, phycobiliproteins, omega-3 (EPA and DHA), omega-6 (γ-linolenic acid), vitamins (A, C, E, B, B1, B2, B3, B5, B6, B9, B12), and phenolic compounds (polyphenolics, *p*-coumaric, and ferulic acid)	Antioxidant, antitumor, anti-inflammatory, immunomodulatory, antifungal, antiviral, as well as radical scavenging properties. Furthermore, it prevents diseases such as atherosclerosis, cardiovascular, and heart related. In addition to this, it has also played key role in healthy skin, blood circulation, preventing clots forming in the blood as well as synthesis DNA and cholesterol in body.	[6]
–	Polysaccharide, protein, essential amino acid, essential fatty acids, minerals, vitamins (vitamin E and carotenoids), phenolics and phycocyanins	Antioxidant, anti-inflammatory, immunostimulatory, antihypertensive, hypoglycemic as well as hypolipidemic properties. Furthermore, it has promoted the activity of natural killer cells as well as improve the growth of beneficial intestinal microbiota.	[150]
–	Carbohydrates, lipids, protein, vitamins, minerals, pigments and phytonutrients	Anticancer, antihypertension, antihypercholesterolemia, antidiabetes, antianemia properties as well as responsible growth of Lactobacilli	[151]
–	Polyunsaturated fatty acid (γ-linoleic acid)	Positive effect on chronic diseases, cancer, diabetes, heart disease, arthritis, Al Zheimer’s disease and inflammatory property	[31]
Spirulina extract 4%	Phenolic and flavonoids compounds as well as high free radical scavenging activity	Antioxidant properties and phagocytosis inhibition	[87]
–	Carbohydrate, protein, vitamins (B1, B2, B3, and E) and minerals (Fe)	Antioxidant, anticancer, antiviral properties as well as control of obesity, allergies, arthritis, inflammation, diabetes, hyperlipidemia, cholesterol and immune system	[48]
–	Polysaccharide, allophycocyanin, carotene, xanthophyll and *C*-phycocyanin	Antioxidant, antivirial, anticancer as well as anti-immunostimulant effects	[33]
–	Polysaccharides, γ-linolenic acid, β-carotene and *C*-phycocyanin	Antioxidant, anticancer, antiviral as well as anti-allergic properties	[152]
–	Acid development and lower pH	Promoted the growth of lactic acid bacteria	[153]
Tablets	Polyunsaturated fatty acids (omega-3 and omega-6 type)	Increased omega-3 omega-6 in human blood lipids as well as cell membrane lipids	[154]
–	Polysaccharide, protein, vitamins, mineral, phycocyanin, and other nutritional supplements	Improved immunity and immune system, activity of superoxide dismutase (SOD) as well as lowering blood lipid level	[41]

– Not reported.

## Data Availability

The data may be shared upon valid request.

## Abbreviations

B	Boron
BGA	Blue-green algae
BMI	Body mass index
Ca	Calcium
CODEX	Codex Alimentarius Commission
Cr	Chromium
Cu	Copper
CVDs	Cardiovascular diseases
DBP	iastolic blood pressure
DHA	Docosahexaenoic acid
dw	Dry weight
EAAs	Essential amino acids
EPA	Eicosapentaenoic acid
FBG	Fasting blood glucose
FDA	Food and Drug Administration
Fe	Iron
GCMS	Gas chromatography–mass spectrometry
HDL	High-density lipoprotein
IBD	Inflammatory bowel disease
IVPD	In vitro digestibility protein
K	Potassium
KCN	Potassium cyanide
LDL	Low-density lipoprotein
Mg	agnesium
Mn	Manganese
Mo	Molybdenum
MUFAs	Monounsaturated fatty acids
Na	Sodium
P	Phosphorous
PUFAs	Polyunsaturated fatty acids
Se	Selenium
SeNPs	Se nanoparticles
WHO	World Health Organization
Zn	Zinc
